# TianTan vaccinia virus-based EBV vaccines targeting both latent and lytic antigens elicits potent immunity against lethal EBV challenge in humanized mice

**DOI:** 10.1080/22221751.2024.2412640

**Published:** 2024-10-10

**Authors:** Xinyu Zhang, Yanhong Chen, Shuhui Wang, Ling Zhong, Zheng Xiang, Xiao Zhang, Shanshan Zhang, Xiang Zhou, Wanlin Zhang, Yan Zhou, Qiuting Zhang, Jingtong Liang, Yanran Luo, Yufei Wang, Ling Chen, Xiaoping Ye, Qisheng Feng, Mu-Sheng Zeng, Ying Liu, Yi-Xin Zeng, Yiming Shao, Miao Xu

**Affiliations:** a State Key Laboratory of Oncology in South China, Collaborative Innovation Center for Cancer Medicine, Guangdong Key Laboratory of Nasopharyngeal Carcinoma Diagnosis and Therapy, Guangdong Provincial Clinical Research Center for Cancer, Sun Yat-sen University Cancer Center, Guangzhou, People’s Republic of China; b State Key Laboratory for Infectious Disease Prevention and Control, National Center for AIDS/STD Control and Prevention, Chinese Center for Disease Control and Prevention, Beijing, People’s Republic of China; c Department of Microbiology and Immunology, School of Medicine, Jinan University, Guangzhou, People’s Republic of China; d College of Pharmacy, Chongqing Medical University, Chongqing, People’s Republic of China; e Department of Hematology, Nanfang Hospital, Southern Medical University, Guangzhou, People’s Republic of China; f Prenatal Diagnostic Center, Guangzhou Women and Children's Medical Center, Guangzhou Medical University, Guangzhou, People’s Republic of China; g Changping Laboratory, Beijing, People’s Republic of China

**Keywords:** Epstein–Barr virus, multi-antigen vaccine, vaccinia virus, T cell response, EBV-induced B cell lymphoma

## Abstract

Epstein–Barr virus (EBV) infection has been related to multiple epithelial cancers and lymphomas. Current efforts in developing a prophylactic EBV vaccine have focused on inducing neutralizing antibodies. However, given the lifelong and persistent nature of EBV infection following primary infection, it is rationalized that an ideal vaccine should elicit both humoral and cellular immune responses targeting multiple stages of the EBV lifecycle. This study used a DNA vector and a TianTan vaccinia virus to express key EBV antigens, including BZLF1, EBNA1, EBNA3B, and gH/gL, to generate multi-antigen vaccines. The multi-antigen vaccine expressing all four antigens and the multi-antigen vaccine expressing BZLF1, EBNA1, and EBNA3B showed comparable protection effects and prevented 100% and 80% of humanized mice, respectively, from EBV-induced fatal B cell lymphoma by activating BZLF1, EBNA1, and EBNA3B specific T cell. The vaccine expressing lytic protein BZLF1 elicited stronger T cell responses and conferred superior protection compared to vaccines targeting single latent EBNA1 or EBNA3B. The vaccine solely expressing gH/gL exhibited no T cell protective effects in our humanized mice model. Our study implicates the potential of EBV vaccines that induce potent cellular responses targeting both latent and lytic phases of the EBV life cycle in the prevention of EBV-induced B cell lymphoma.

## Introduction

Epstein–Barr virus (EBV) is one of the most widespread human viruses. After primary infection, most individuals carry EBV asymptomatically. Yet, its persistency has a remarkable potential to cause diseases in hosts, including Burkitt lymphomas (BL), Hodgkin lymphoma (HL), gastric carcinomas (GC), and nasopharyngeal carcinomas (NPC), as well as multiple sclerosis (MS). Following primary infection, EBV establishes a lifelong persistent infection in host memory B cell, which can lead to occasional reactivations. Robust EBV-specific memory T cell expansion against both lytic and latent antigens inhibits the development of EBV-immortalized B cells in immune-competent hosts. EBV-positive people with compromised immune systems are susceptible to viral reactivation and malignant transformation. Furthermore, EBV-positive patients who receive immunosuppressive medication following organ or stem cell transplantation are very susceptible to develop post-transplant lymphoproliferative disease (PTLD), a B-cell lymphoproliferative illness (LPD) that often results in death. However, there are still no licensed EBV vaccines for these EBV-LPD patients.

The current therapy choices for these patients are limited. It is important to develop EBV vaccines that can control EBV replication and prevent EBV-induced LPD, potentially benefiting the patients at high risk of developing LPD. Previous prophylactic EBV vaccine studies have mainly focused on viral envelope glycoproteins, gp350, gH/gL, gB, and gp42 [1–4]. However, the evidence demonstrating that neutralizing antibodies can prevent associated diseases in those who have already been infected is limited [5]. Therefore, to prevent diseases in EBV carriers, it is crucial to broaden the antigenic diversity of the EBV vaccine beyond membrane proteins that elicit neutralizing antibodies.

T cells play an important role in controlling viral load and disease progression [6, 7]. EBV has a complex life cycle, transitioning between latent and lytic cycles by expressing distinct sets of viral genes [8]. Once infected with EBV, healthy carriers establish memory T cells against a broad range of EBV proteins to control EBV infection [9]. Intermittent reactivation of EBV in the oropharynx of healthy individuals establishes hierarchical T cell responses, with most EBV-specific CD8^+^ T cells identified in the periphery being specific for immediate early proteins BZLF1 and BRLF1, to a lesser extent against early antigens and a few against late antigens [9]. For latent antigens, the dominant CD8^+^ T cell responses are against the EBNA3 proteins, with sub-dominant responses also present against EBNA1, EBNA2, and LMP2 in healthy individuals, and immune responses against EBNA-LP and LMP1 are rare [10]. When T cell immunity is compromised, e.g. under the condition of HIV infection or post-transplant immunosuppression, the risk of developing EBV-associated lymphomas increases [6].

The production of EBV virion to colonize B cells immediately after EBV infection is thought to be important for LCL formation *in vivo* and *in vitro* [11]. This initial seeding of infected B cells is followed by B cell transformation and the uncontrolled proliferation of the transformed B cells [10, 11]. T cells that target the initial EBV colonization have the working window to effectively prevent the formation EBV-LPD. Hence, EBV proteins expressed in the early stages of transforming B cells and/or in the LCLs are considered potential targets in our vaccine design against EBV-induced B cell lymphoma.

Among the key EBV proteins involved in the early stages of B cell transformation, immediate early lytic BZLF1 and latent EBNA1 and EBNA3B have emerged as promising vaccine targets. BZLF1 has been documented as the first antigen recognized by the human immune response upon primary infection and is essential to drive B-cell immortalization *in vitro* [12, 13]. The BZLF1-specific T cells prevent the development of EBV-induced B cell lymphoma *in vitro* [14]. EBNA1 is an oncogenic protein that promotes the transformation of infected B cells and plays a critical role in tumorigenesis [15]. EBNA1 is also the only protein expressed in all EBV-infected cells, except in the latency 0 stage [15]. Adoptive transfer of T cells targeting EBNA1 was proved to be effective in the therapy for HL [16–18]. EBNA3B belongs to the EBNA3 family, which also includes EBNA3A and EBNA3C. EBNA3A and EBNA3C play a role in B cell transformation and are thus considered oncogenic [19]. However, EBNA3B negatively regulates EBNA3A and EBNA3C, exhibiting tumour-suppressive function [19]. EBNA3B-specific T cells can effectively inhibit B cell transformation and LCL outgrowth *in vitro* [13]. gH/gL is the core component of EBV fusion machinery and is an important target of EBV neutralizing antibodies [20], while there has been relatively few research on gH/gL-specific T cell response. Based on a few researches, gH/gL T cell immunity was relatively weak compared to the BZLF1, EBNA1, and EBNA3B [1]. Thus, we used gH/gL antigen in the humanized mouse experiments for the comparison of T cell responses and vaccine protection induced by T cell responses.

Considering the breadth of EBV-specific immune responses observed in healthy individuals and the expression of the multiple latent proteins and immediate early proteins in the early stage of EBV-induced B cell transformation. We selected BZLF1, EBNA1, EBNA3B, and gH/gL as our potential vaccine immunogen. The broad spectrum multi-antigen vaccine may be superior to targeting a single phase in the design of EBV vaccine to inhibit EBV-induced B cell transformation, LCL outgrowth, and the development of EBV-LPD.

The replication-competent TianTan Vaccinia virus (rTTV) has been utilized as a vaccine for smallpox prevention in China, with a cumulative vaccination of hundreds of millions of people, showing good efficacy and safety [21]. Following the eradication of smallpox, rTTV was used as a vector for developing various recombinant vaccines [22, 23]. rTTV and its relatives, such as the Ankara vaccinia virus, have been demonstrated to elicit robust and long-lasting humoral and cellular immunity [24, 25]. Previous studies have shown the efficiency of the heterologous DNA prime-rTTV boost regimen for vaccination [23, 26]. To minimize heterologous immune responses against vaccine backbones, we used this “prime-boost” vaccination regimen that sequentially delivers DNA and rTTV vectors containing the antigens of interest.

In this study, we utilized TianTan vaccinia virus as a vector to express BZLF1, EBNA1, EBNA3B, and gH/gL from the M81 strain and designed multi-antigen EBV vaccines, and the specific aim of our study is to prevent EBV-induced B cell lymphoma. We evaluated the protective effects of vaccines over lethal EBV challenge in a humanized mouse model. Our results showed that the multi-antigen vaccine completely prevented the development of EBV-induced B cell lymphomas and protected humanized mice from lethal EBV challenge. Furthermore, we studied the potential protective mechanisms of multi-antigen vaccines and compared the protective efficacy of multi-antigen vaccines and single-antigen vaccines in inhibiting EBV infection, reducing viral load, and preventing associated tumours.

## Material and methods

### Ethics statements

All animal experiments were performed under protocols approved by the Sun Yat-sen University Cancer Center's Animal Care and Use Committee. The human peripheral blood mononuclear cells (PBMCs) used for humanized mice reconstitution were collected from healthy blood donors. This study was approved by the Institutional Ethics Committee of the Sun Yat-sen University Cancer Center, located in Guangdong, China.

### Cells lines

All cell lines were cultured at 37°C in a humidified atmosphere with 5% CO_2_. CEF, HEK-293 T, and BHK-21 cell lines were cultured in Dulbecco's modified Eagle medium (DMEM; Invitrogen) supplemented with penicillin (100 U/ml), streptomycin (100 μg/ml), and 10% fetal bovine serum (FBS; Invitrogen). HNE1 and Akata B cells were cultured in RPMI-1640 medium supplemented with penicillin (100 U/ml), streptomycin (100 μg/ml), and 10% fetal bovine serum (FBS; Invitrogen). Akata-EBV and CNE2-EBV cells were cultured in RPMI-1640 medium supplemented with penicillin (100 U/ml), streptomycin (100 μg/ml), G418 (700 μg/ml; MP Biomedicals), and 10% fetal bovine serum (FBS; Invitrogen).

### Gene synthesis and plasmid construction

The coding sequences of BZLF1 (amino acids 53–227, Genbank AGZ95183.1 with the residues C^171,189, and 222^ mutated to S), EBNA1 gene fragment (amino acids 391–645, Genbank AHF72808.1 with the residues E^448 and 451^ mutated to A), EBNA3B (amino acids 1-160, 166-869, and 873-938, Genbank AGZ95180.1), and gH/gL gene fragment (the full-length sequence of gL [Genbank AGZ95188.1] linked to the amino acids of 19–679 of gH [Genbank AGZ95211.1] by (G4S)_3_ from EBV M81 strain) were synthesized, codon optimized for human cells, and subsequently cloned into pDRVI3.0 and pSC65-GFP plasmids. M81 is the most common EBV strain found in South China. Both the B95-8 and M81 strains belong to the type 1 strain of EBV. M81 is distinguished from the prototype B95-8 EBV strain by its ability to replicate lytic spontaneously and generate high-titre viruses [27].

The full-length BALF5 gene was PCR-amplified from EBV M81 BAC and subsequently inserted into the pUC19 vector. The pUC19 vector was then utilized to create a standard curve for quantifying EBV DNA copy numbers.

### Construction of recombinant rTTV vaccines

CEF cells (1 × 10^6^ cells) were infected with wild rTTV at a multiplicity of 0.1 PFU (Plaque Forming Unit) per cell and transfected two hours later with 10 μg of linearized plasmids pSC65-GFP. These plasmids contained BZLF1, EBNA1, EBNA3B, or gH/gL and were transfected using Lipofectamine 3000 reagent following the manufacturer's recommendations (Invitrogen). The transfection medium was then replaced with fresh DMEM medium supplemented with 10% FBS. After 48 hours post-infection (hpi), the cells were collected, lysed three times using freeze–thaw cycling, and used for recombinant virus screening. Recombinant rTTV vaccines that contained the antigen DNA fragments and co-expressed the GFP marker gene were selected through consecutive rounds of plaque purification in BHK-21 cells. In each round of purification, the isolated plaques were expanded in BHK-21 cells for two days, and the crude viruses were used for the next round of plaque purification. The recombinant rTTV vaccines were grown in BHK-21 cells, purified through 30% (w/v) sucrose cushions, and titrated using the plaque forming assay.

### Detection of rTTV DNA in tissue

DNA was extracted from tissues (20 mg) of BALB/c mice using commercial DNA extraction kits (Omega). Primers TK-L (5'-TGATTAGTTTGATGCGATTC-3’) and TK-R (5'-TGTCCTTGATACGGCAG-3’) annealing in the TK flanking sequences were used for PCR analysis to rTTV DNA residues.

### Analysis of recombinant rTTV vaccine growth

To determine the effect of antigenic gene insertion into the rTTV genome on virus replication, we compared recombinant rTTV vaccines with parental wild-rTTV. BHK-21 cells and HEK-293 T cells were grown in 12-cell plates and were infected in duplicate at 0.01 PFU/cell with wild-rTTV or with the different recombinant rTTV vaccines. Following virus infection for two h at 37°C, the medium was removed, and the infected cells were washed with phosphate-buffered saline (PBS) three times and incubated with DMEM containing 2% FBS at 37°C in 5% CO_2_. Cells were collected, frozen, and thawed three times at different times post-infection. Virus titres were determined by the plaque forming assay.

### Expression of antigen proteins from recombinant rTTV vaccines by Western blot analysis

BHK-21 cells were infected at 0.1 PFU/cell with wild-rTTV or recombinant rTTV vaccines. Following virus infection for two hours at 37°C, the medium was removed, and the infected cells were washed with PBS three times and incubated with DMEM containing 2% FBS for 8 hours. Cells were lysed in 1×SDS-PAGE loading buffer, and cell extracts were fractionated by 10% SDS-PAGE and analyzed by Western blotting.

### Expression of antigen proteins from recombinant rTTV vaccines by confocal immunofluorescence microscopy analysis

BHK-21 cells were cultured on glass coverslips until they reached a confluence of 70%. Then, they were infected with either wild-rTTV or recombinant rTTV vaccines at a concentration of 0.1 PFU/per cell. After two hours of virus infection at 37℃, the medium was removed, and the infected cells were washed three times with PBS. Subsequently, the cells were incubated with DMEM containing 2% FBS for 48 hours. The cells were washed again with PBS and fixed with 4% paraformaldehyde (PFA) at room temperature for 10 min. Following fixation, the cells were incubated in a blocking solution (5% BSA, 0.5% Triton X-100 in PBST) for one hour at room temperature and then incubated overnight at 4°C with primary antibodies. After being washed three times with PBST, the cells were incubated with secondary antibodies in the dark at room temperature. Once again, the cells were washed thrice with PBST and then incubated with DAPI for 5 min. Finally, images were captured using an FV1000 OLYMPUS microscope.

### Genetic stability of recombinant rTTV vaccines by protein expression analysis

The antigen genetic stability of recombinant rTTV vaccines was analyzed as previously described. BHK-21 cells were infected with different recombinant TTVs vaccines at a 0.1 PFU/cell concentration for 48 hours. Cells were collected and subjected to three freeze–thaw cycles for lysis. The lysed cells were then used for a subsequent round of infection. The above procedure was repeated 12 times. The expression of antigen proteins was detected using Western blotting.

### Time course expression of antigens proteins from recombinant rTTV vaccines

BHK-21 cells or HEK-293 T cells were cultured in 6-well culture plates and were infected at 1 PFU/cell with wild-rTTV or recombinant TTVs vaccines. After different hours of post-infection, the cells were collected and lysed with a 1×SDS-PAGE loading buffer. Cell extracts were fractionated by 10% SDS-PAGE and analyzed by Western Blotting.

### EBV production

CNE2-EBV cells and Akata-EBV cells carrying the Akata-EBV-GFP BAC were used to produce EBV derived from epithelial cells (CNE2-EBV-GFP) and B cells (Akata-EBV-GFP), respectively. To produce B-cell tropic GFP reporter viruses, CNE2-EBV-GFP cells were treated with 20 ng/ml 12-O-tetradecanoylphorbol 13-acetate (TPA; Beyotime) and 2.5 mM sodium butyrate (NaB; Sigma Aldrich) for 12 hours, the cells were washed twice with PBS and replaced with fresh complete medium. To produce epithelial cell tropic GFP reporter virus, Akata-EBV-GFP cells were suspended at 4 × 10^6^ cells/ml in RPMI medium. Goat anti-human IgG (100 μg/ml final concentration; Tianfun Xinqu Zhenglong Biochem. Lab) was added for six hours, and the cells were then diluted to 2 × 10^6^ cells/ml in RPMI containing 5% FBS and incubated. After a 96-hour culture period, the cell supernatant was collected, centrifuged, and filtered using a 0.8 μm filter. The viruses were subsequently concentrated 100-fold through centrifugation at 50,000×g for three hours and resuspended in serum-free RPMI 1640. Finally, the viruses were stored at – 80°C.

### Establishment of EBV-lymphoblastoid cell lines (EBV-LCLs) *In Vitro*

Briefly, huPBMC were infected with Akata and cultivated in RPMI 1640 supplemented with 10% heat-inactivated fetal bovine serum and 0.4 μg/ml cyclosporine. The LCLs were stable and ready for use in later studies after around 4 or 5 weeks.

### Determination of EBV gene expression in primary B cell and LCL by qRT-PCR

Total RNA was extracted, purified, and used to synthesize cDNA. The obtained cDNA was used to perform quantitative real-time PCR for BZLF1, EBNA1, EBNA3B, gL, gH, and GAPDH. The primers used are listed in Table 2.

### RNA-seq analysis

Normalized EBV gene expression levels (GEO, GSE125974) are displayed by a heat map. This data included EBV gene expression in B cells on day 0, day 2, day 4, day 7, day 14, day 21, and day 28 after EBV infection.

### Immunizations in BALB/c mice

All animal experiments were performed under protocols approved by the Sun Yat-sen University Cancer Center Animal Care and Use Committee. Four-week-old female BALB/c mice were prime immunized at weeks zero, two, and four with 200 µg total DNA vaccines (per antigen is 50 μg) followed by in vivo electroporation, and boost immunized at week six with 5 × 10^6^ PFUs total recombinant TTVs vaccines (per antigen is 1.25 × 10^6^ PFUs) at a total volume of 100 µl. Mice were immunized via intramuscular injection split into two 50 µl doses between both rear legs. Blood was collected retro-orbitally two weeks after the third DNA vaccine immunization and three weeks after the final vaccination. Blood was collected in tubes containing 1/10 the volume of citrate. Serum was separated from whole blood via centrifugation and then heat-inactivated at 56°C for 30 min.

### Antibody titre and avidity testing

BZLF1, EBNA1, EBNA3B, or gH/gL (100 ng/well) was plated onto a 96-well ELISA plate (Corning) overnight at 4°C. After being washed with TBST three times, the ELISA plates were coated with a blocking buffer (5% blotting-grade milk powder and 3% BSA in DPBS with 0.05% Tween 20) and incubated for two hours at 37°C. Then, the ELISA plates were washed with PBST three times. The ELISA plates were incubated with serially diluted serum samples (starting from 100, three-fold) for two hours at room temperature (RT), followed by five washes. The presence of total BZLF1, EBNA1, EBNA3B, and gH/gL-specific mouse IgG and IgM were detected using HRP-conjugated anti-mouse (1:5000) (Promega) for 1 hour at room temperature (RT). The ELISA plates were washed five times and developed using TMB (Sangon Biotec), and the reaction was stopped using 1 N phosphoric acid solution. Plates were read at a wavelength of 450 nm within 30 min using a microplate reader (Molecular Devices).

The antibody avidity index was determined by diluting the immune serum to a concentration corresponding to an OD_450_ = 3.0 in an endpoint ELISA. Serum samples were then reacted with antigen-coated plates for two hours at 37°C. After washing, 1.5 M chaotropic reagent sodium thiocyanate were added (100 μl/well). The plates were washed and dried for 20 min, and then the remaining plate-bound antibodies were measured. The avidity index is determined by the percentage of antibodies that remained bound at the antigen coat after the treatment with chaotropic reagent sodium thiocyanate: The antibody avidity index (%) = OD_450_ with NaSCN/ OD_450_ without NaSCN ×100.

### Neutralization assays

All serum samples used in this assay were treated at 56°C for 45 min to inactivate the complement. For B cell neutralization, mouse serum was serially diluted two-fold (starting from a 1:20 dilution) and incubated with 20 μl of Akata-EBV-GFP (diluted to achieve an infection frequency of 20-30% at the final dilution) for two hours at 37°C. Subsequently, the mixtures were added to 1 × 10^4^ EBV-negative Akata B cells in 96-well plates and incubated for three hours at 37°C. For epithelial cell neutralization, mouse serum was also serially diluted two-fold (starting from a 1:20 dilution) and incubated with 100 μl of Akata-EBV-GFP (diluted to achieve an infection frequency of 2-5% at the final dilution) for two hours at room temperature. The mixture was then added to 1 × 10^4^ HNE1 epithelial cells/well in 48-well plates and incubated for three hours at 37°C. After that, the free EBV viruses in the culture medium in both assays were removed by washing once with PBS. The cells were further cultured in fresh RPMI 1640 with 10% FBS for 48 hours, washed once with PBS, and the infection rate was determined by detecting and analyzing the numbers of GFP-positive cells using CytoFLEX (Beckman Coulter) and FlowJo software × 10 (Tree Star). Negative controls for the B cell and epithelial cell neutralization assays were EBV-negative Akata B cells and HNE1 epithelial cells, respectively. Positive controls for the B cell and epithelial cell neutralization assay were EBV-negative Akata B cells and HNE1 epithelial cells incubated with EBV without antibodies. The neutralizing efficiency of antibodies was calculated using the following formula: neutralization % = 1 – (the percent of infected cells with serum/the percent of infected cells without serum) × 100%. The neutralization curve was fitted using the log (inhibitor) versus response-variable slope (four parameters) analysis in Prism 9.2.0. The half-maximal inhibitory plasma dilution (ID_50_) was interpolated from the curve in Prism 9.2.0.

### Generation of humanized mice

SCID beige mice (CB17.B6-*Prkdc^scid^Lyst^bg^*/Crl) were acquired from Charles River and housed in Sun Yat-sen University Cancer Center's Animal Care and Use Committee. Human peripheral blood mononuclear cells (hPBMCs) were isolated from buffy coat preparations obtained from healthy donors at the Guangzhou Red Cross to establish humanized mouse models. Four-week-old female SCID beige mice were irradiated (1 Gy) with an RS2000 irradiator (RAD Source) and intravenously injected with Clodronate Liposomes (YEASEN; 500 μg/mouse) one day before transplantation. After 24 h, each mouse was intraperitoneally transplanted with 1.5 × 10^7^ hPBMCs. Typically, hPBMCs from one buffy coat were utilized to generate 10–15 humanized mice. Daily monitoring was conducted following transplantation to observe graft-versus-host disease symptoms, including weight loss, temperature changes, and diarrhea. The immune reconstitution of humanized mice was evaluated at specified time points by immunophenotyping circulating lymphocytes using antibodies with indicated dilutions: hCD45-APC-CY7 (1:200), hCD3-FITC (1:200), hCD4-PB (1:200), hCD8-PercP-CY5.5 (1:200), hCD19-APC (1:250), hCD2-APC (1:100), and mCD45-BV510 (1:200). Seven weeks post-challenge, mice were euthanized and single-cell suspensions of peritoneal fluid were collected and immunophenotyping of B cells using hCD45-APC/CY7 (1:100), CD19-APC (1:100), hCD38-BV510 (1:100), and hCD24-PC5.5 (1:100), or T cells using hCD45-APC/CY7 (1:100), mCD45-BV510 (1:100), hCD4-pacific blue (1:100), hCD8-PC5.5 (1:100), hCD137-APC (1:100). All animal experiments were conducted following protocols approved by the Animal Care and Use Committee of Sun Yat-sen University Cancer Center.

### Immunization and EBV challenge in humanized mice

Four weeks post-transplantation of human peripheral blood mononuclear cells (hPBMCs), humanized mice were primed intraperitoneally with 20 μg DNA vaccines (5 μg per antigen) by polyplus *in vivo-*jetPEI™ transfer reagent. Two weeks after priming, a boost vaccination of 5 × 10^2^ PFUs total rTTV vaccines (1.25 × 10^2^ PFUs/per antigen) was administered by intraperitoneal injection. Two weeks after the boost immunization, the mice were intraperitoneally injected with a dose of Akata-EBV equivalent to 100,000 infectious units, as determined by infecting Raji cells. Starting from one week post-challenge (day seven), blood samples were collected weekly to detect the presence of EBV DNA in whole blood and the immunophenotype of circulating lymphocytes. Mice were weighed once a week. They were euthanized if their weight fell below 80% of their initial weight or if they exhibited severe GVHD reactions, including hair loss and spinal curvature.

### Detection of EBV DNA in blood and tissues

DNA was extracted from the peripheral blood (50 μl) or tissues (20 mg) of humanized mice using commercial DNA extraction kits (Omega). EBV copy numbers were then detected by real-time polymerase chain reaction (RT–PCR) using these primers (F: 5'-GGTCACAATCTCCACGCTGA-3’; R: 5'-CAACGAGGCTGACCTGATCC-3’) to amplify a fragment of BALF5. The copy number in extracted DNA was determined by interpolating from the standard curve with a serially diluted plasmid of pUC19-BALF5.

### H&E staining, immunohistochemistry, and in situ hybridization

Tissue samples were fixed in 10% formalin and embedded in paraffin. The treated samples were stained with hematoxylin and eosin (H&E). EBERs were detected by in situ hybridizations using an EBER detection kit (ZSGB-BIO) according to the manufacturer's instructions. Immunostaining of human B cells was performed using an hCD20 antibody (Abcam) at a 1:200 dilution.

### Intracellular cytokine staining assay

BALB/c mice spleens were removed under aseptic conditions and stimulated with BZLF1 (Miltenyi Biotec), EBNA1(Miltenyi Biotec), and EBNA3B (JPT Peptide Technologies GmbH) peptide pools (5 μg/ml) or gH/gL protein (5 μg/ml). Brefeldin A (10 μg/ml) and Monesin (1 μM) were added after 2 hours of incubation, and cells were incubated for an additional four hours. Then, the cells were incubated and stained with Live/Dead-BV510 (Thermo) for 20 min at room temperature (RT) in the dark and washed twice in PBS. Cells were blocked for 30 min at 4°C in Fc-blocking solution (5 μg/ml of anti-mCD16/CD32 mAb, eBioscience, USA) and washed twice in PBS. Cells were stained with mCD45-APC/CY7 (BioLegend), mCD3^+^-FITC (BioLegend), mCD8-APC (BioLegend), and mCD4^+^-AF700 (BioLegend) surface markers. Cells were fixed and permeabilized for 30 min at 4°C with a permeabilization buffer (Thermo Fisher Scientific, USA). Cells then were stained with anti-mouse IFN-γ-PE/Cy7 antibody (BioLegend), anti-mouse TNF-α-BV421 antibody (BioLegend) and anti-mouse IL-2-PE antibody (BioLegend) at room temperature for 60 min in the dark. Cells with no stimulation were used as a negative control, while cells stimulated with phorbol myristate acetate (PMA)-ionomycin (Sigma) were used as a positive control.

Humanized mice blood was treated with 1 ml of red blood cell lysis buffer (BioLegend) at room temperature for 10 min. Then, the cells were centrifuged at 300×g, washed twice with PBS, resuspended in 100 μl of RPMI 1640 medium containing 10% FBS with anti-human CD28 (1 μg/ml, clone CD28.2), anti-human CD49d (1 μg/ml, clone 9F10), these cells were stimulated with BZLF1 (Miltenyi Biotec), EBNA1(Miltenyi Biotec), and EBNA3B (JPT Peptide Technologies GmbH) peptide pools (5 μg/ml) or gH/gL protein (5 μg/ml). Brefeldin A (10 μg/ml) and Monesin (1 μM) were added after two hours of incubation, and cells were incubated for an additional four hours. Cells were transferred to 4°C overnight and stained the next day for expression of cytokines. Then, the cells were incubated and stained with Live/Dead-BV510 (Thermo) for 20 min at room temperature (RT) in the dark and washed twice in PBS. Cells were blocked for 30 min at 4°C in Fc-blocking solution (5 μg/ml of anti-mCD16/CD32 mAb and anti-hCD16/CD32 mAb eBioscience, USA) and washed twice in PBS. Cells were stained with anti-human CD45-PE (BioLegend), anti-human CD3-AF700 (BioLegend), anti-human CD8-APC/CY7 (BioLegend), anti-human CD4-PercP/Cy5.5 (BioLegend). Cells were fixed and permeabilized for 30 min at 4°C with a permeabilization buffer (Thermo Fisher Scientific, USA). Permeabilized cells were stained with ICS antibodies to anti-human IFN-γ-AF647 (BioLegend), anti-human TNF-α-BV421 (BioLegend), anti-human IL-2-FITC (BioLegend). Cells were washed twice with perm/wash buffer. Cells with no stimulation were used as a negative control, while cells stimulated with phorbol myristate acetate (PMA)-ionomycin (Sigma) were used as a positive control.

The assays were performed with a CytoFLEX (Beckman Coulter), and the data were analyzed using FlowJo software v.10 (TreeStar).

### Statistical analysis

All statistical analyses were conducted with GraphPad Prism version 8. Statistical tests are indicated in figure legends.

## Results

### Construction and characterization of recombinant DNA-based and rTTV-based multi-antigen EBV vaccine

By analyzing public RNA-seq data (Figure S1A) and qRT-PCR (Figure S1B) [28]. We found that immediate early lytic genes such as BZLF1 and BRLF1 and latent genes such as EBNA1, EBNA2, EBNA-LP, and EBNA3B were expressed at the very early stages after EBV infection and throughout LCL formation (from Day 2 to Day 28). From these genes, we selected EBNA1, EBNA3B, and BZLF1 genes because they are reported to be the targets of strong T cell responses [10]. We expect to induce their specific T cell immunity to recognize and kill recently infected B cells and effectively inhibit the transformation and proliferation of B cells, therefore preventing EBV-LPD formation at the early stage.

**Figure 1.**
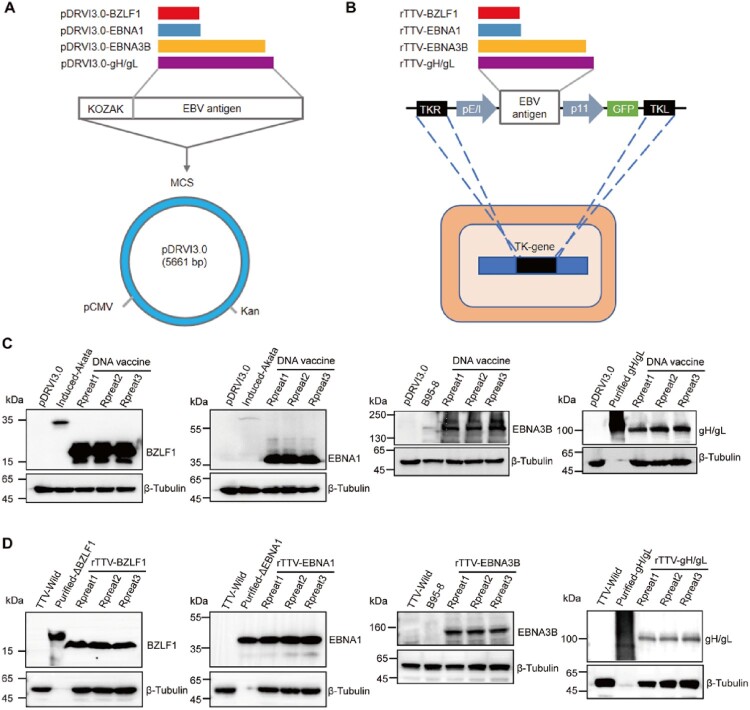
Design and generation of DNA – and rTTV-vector-based multi-antigen EBV vaccine (A) Diagram of multi-antigen EBV DNA vaccine comprising four plasmids encoding EBV antigens derived from the M81 strain. (B) Diagram of multi-antigen EBV rTTV vaccine containing four rTTV encoding EBV antigens derived from the M81 strain. Antigen genes were inserted into the essential region between TKR and TKL under the PE/I promoter. Western Blot analysis of multi-antigen EBV DNA vaccine expression in HEK-293 T (C) and multi-antigen EBV rTTV vaccine expression in BHK-21(D). HEK-293 T cells were transfected with recombinant DNA vaccines or an empty vector. At 48 hours post-infection, cells were lysed and analyzed by Western blotting. Similarly, BHK-21 cells were infected at 1 PFU/cell with recombinant rTTV vaccines or wild-type rTTV. At 24 hours post-infection, cells were lysed and analyzed by Western blotting. We used two mouse monoclonal antibodies against BZLF1 and EBNA1, a rabbit monoclonal antibody against gH/gL, and a sheep polyclonal antibody against EBNA3B. A rabbit polyclonal antibody against β-Tubulin was used as a load control of the quantity of host-cell protein. Arrows on the right indicate the BZLF1, EBNA1, EBNA3B, and gH/gL proteins. The sizes of standards (in kDa) are indicated on the left.

We next constructed the recombinant DNA-based and rTTV-based multi-antigen EBV vaccines by inserting BZLF1, EBNA1, EBNA3B, and gH/gL encoding sequences (M81 strain, KF373730.1) into the *Sac* I site of pDRVI3.0 (Figure 1A) and the thymidine kinase (TK) locus under the pE/I promote of the rTTV (Figure 1B). Antigen modifications were made to remove repetitive sequences and nuclear localization sequences from EBNA1 and EBNA3B and to ablate potential effects regions inherent to the proteins BZLF1 and EBNA1 while preserving the T-cell epitope-rich domain (Figure S2A). The workflow of the recombinant rTTV-based vaccine construction is illustrated in Figure S2B. HEK-293 T cells were transfected with each recombinant DNA-based vaccine to test the expression of BZLF1, EBNA1, EBNA3B, or gH/gL. Western blotting of the lysates from transfected cells revealed bands approximately to the molecular weight of the predicted antigens (Figure 1C). Furthermore, antigen expression was observed by confocal microscopy (Figure S3A). We detected similar results using western blotting and confocal microscopy after each recombinant rTTV-based vaccine infected BHK-21 cells (Figure 1D and Figure S3B). We also used western blots and plaque experiments to confirm that the insertion of antigen genes did not impair virus vector replication (Figures S4A-S4C).

### The heterologous prime-boost multi-antigen EBV vaccine induced robust antigen-specific immune responses in BALB/c mice

**Figure 2.**
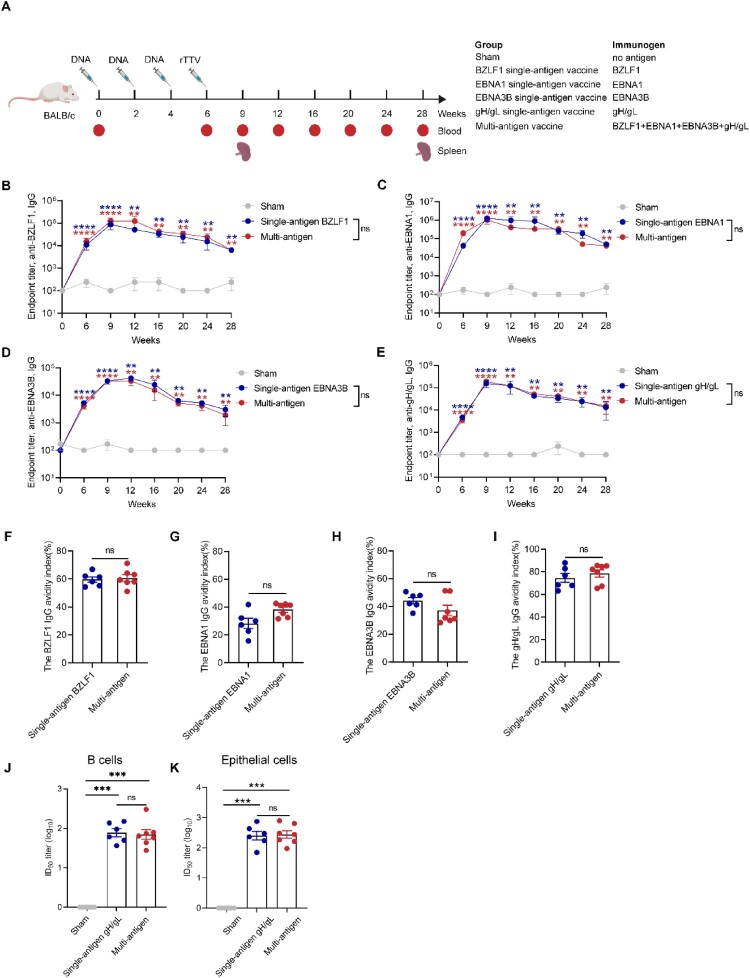
Heterologous prime-boost vaccination with multi-antigen EBV vaccine induces humoral immune responses in BALB/c mice. (A) Schematic representation of immunization groups and regimes. The figure was created from Biorender.com. BALB/c mice were immunized intramuscularly with single-antigen or multi-antigen DNA vaccines (50 μg per antigen) at weeks 0, 2, and 4 and single-antigen or multi-antigen rTTV vaccines (1.25 × 10^6^ PFUs per antigen) at week 6. Blood was collected at weeks 0, 6, 9, 12, 16, 20, 24 and 28. Spleens were collected at weeks 9 and 28. Mice that received sham immunizations served as controls. Sera were collected from mice immunized with multi-antigen vaccine at weeks 0, 6, 9, 12, 16, 20, 24, and 28 and determined the specific IgG of BZLF1 (B), EBNA1 (C), EBNA3B (D), and gH/gL (E) measured by ELISA. Comparison of antigen-specific IgG (B-E), antibody avidity (F-I) and EBV B-cell – and epithelial-cell-neutralizing titres in serum (J and K) between the single-antigen or the multi-antigen vaccines on 2 weeks after the final immunization (week 9). IgG avidity index (%) measured by ELISA using NaSCN: OD_450_ with NaSCN/OD_450_ without NaSCN ×100. Half maximal inhibitory dilution fold (ID_50_) was calculated by sigmoid trend fitting. Each dot represents an individual mouse's reciprocal half-maximal inhibitory dilution (ID_50_) titre. 0 indicates that no neutralizing ability was detected at the tested serum dilutions. Each dot represents an individual mouse. The data are shown as the mean ± standard error of the mean (SEM). Before the experiment, Sham n = 11, BZLF1 single-antigen vaccine n = 11, EBNA1 single-antigen vaccine n = 11, EBNA3B single-antigen vaccine n = 11, gH/gL single-antigen vaccine n = 11, and multi-antigen vaccine n = 12. After week 9, there are 5 mice in every group. *P*-values were determined by Mann-Whitney rank-sum test (ns, *P *≥ 0.05; *P < 0.05; **P < 0.01; ***P < 0.001; ****P < 0.0001).

In our previous vaccine studies, we determined that the heterogenic immunization strategy of three doses of DNA vaccine (50 μg/per antigen) and one dose of rTTV vaccine (1 × 10^6^ PFUs/per antigen) boost immunization induced more robust cellular and humoral immune responses than DNA vaccine or rTTV homologous vaccination strategy alone [23, 29, 30]. Additionally, several researches have documented the benefits of implementing DNA prime/rTTV boost heterogenous immunization strategy for vaccinia virus-based vaccines [31–33]. Hence, in this research, we selected this vaccination strategy to examine the safety, immunogenicity, and antigenic competition of our EBV multi-antigen vaccine. Detailed immunization strategies are shown in Figure 2A.

The heterologous prime-boost multi-antigen vaccine had negligible effects on mice survival (Figure S5A), body weight (Figure S5B), and gross organ anatomy (Figure S5C). Some mice were euthanized three weeks after the final immunization, and their brain, heart, liver, spleen, lung, and intestine tissues were collected. Hematoxylin and eosin (H&E) staining and polymerase chain reaction (PCR) showed no detectable tissue damage or residual rTTV virus in these organs (Figures S5D and S5E). These results demonstrate that the heterologous prime-boost multi-antigen vaccine is safe in mice.

We evaluated serum IgG antibodies to BZLF1, EBNA1, EBNA3B, and gH/gL using enzyme-linked immunosorbent assay (ELISA). Antigen-specific antibodies against BZLF1, EBNA1, EBNA3B, and gH/gL were detected in the serum of mice two weeks after the final immunization with the multi-antigen DNA vaccine (Figure 2B-E). Higher-titre antigen-specific antibodies were detected in the serum of mice three weeks after vaccination with the multi-antigen rTTV vaccine (Figure 2B-E). Moreover, the antigen-specific antibodies in mice immunized with the heterologous prime-boost multi-antigen vaccine could be detected for more than 5 months without further boosting. The antibody titres (Figure 2B-E) and antibody avidity (Figure 2F-I) against each antigen remained at similar levels when the four antigens were administered individually or in combination. No immune competition was observed at the current dose used for the vaccination.

gH/gL is the component of the core fusion machinery necessary for EBV infection in both B and epithelial cells [34]. Multiple anti-gH/gL human antibodies have been identified that potently block EBV infection in both cell types [20, 35, 36]. The neutralizing activity against EBV infection of serum from animals immunized with the single-antigen gH/gL vaccine and the multi-antigen vaccine was evaluated in both B cells and epithelial cells. Both vaccines elicited similar levels of potent neutralizing antibodies that blocked virus entry into Akata B cells and HNE1 epithelial cells (Figure 2J and K).

**Figure 3.**
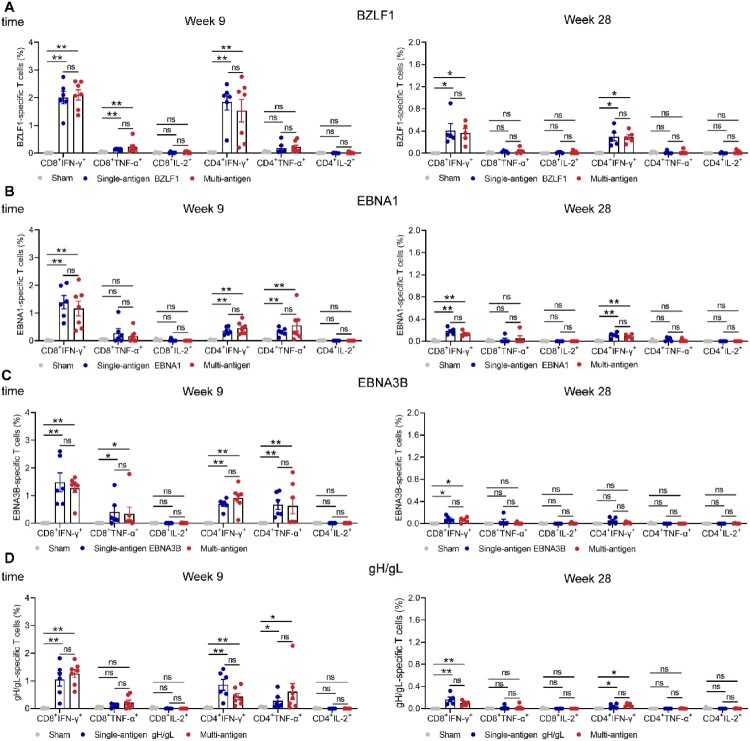
Heterologous prime-boost vaccination with multi-antigen EBV vaccine induces cellular immune responses in BALB/c mice. Splenocytes were stimulated with BZLF1, EBNA1, and EBNA3B peptide pools (5 μg/ml) and gH/gL protein (5 μg/ml) 9 and 28 weeks after the first immunization and IFN-γ, TNF-α, or IL-2 production by CD8^+^ and CD4^+^ T cells was assessed by flow cytometry. Percentages of BZLF1 (A), EBNA1 (B), EBNA3B (C), and gH/gL-specific (D) CD8^+^ and CD4^+^ T cells producing IFN-γ, TNF-α, and IL-2 are shown. Each dot represents an individual mouse. The data are shown as the mean ± standard error of the mean (SEM). Sham n = 6, BZLF1 single-antigen vaccine n = 6, EBNA1 single-antigen vaccine n = 6, EBNA3B single-antigen vaccine n = 6, gH/gL single-antigen vaccine n = 6, and multi-antigen vaccine n = 7 at 3 weeks after the final immunization. At week 28, there are 5 mice in every group. *P*-values were determined by Mann-Whitney rank-sum test (ns, *P *≥ 0.05; *P < 0.05; **P < 0.01; ***P < 0.001; ****P < 0.0001).

To examine T cell durable responses, the splenocytes of immunized mice were collected three and twenty-two weeks after the final multi-antigen rTTV vaccine immunization and stimulated with overlapping peptide pools specific to BZLF1, EBNA1, EBNA3B, and gH/gL protein. The frequency of antigen-specific CD8^+^ T cells and CD4^+^ T cells expressing type 1 (Th1) immune response cytokines (interferon-γ [IFN-γ], tumour necrosis factor-α [TNF-α] and interleukin-2 [IL-2]) were measured by the intracellular cytokine staining (ICS) assay (Figure S6). The heterologous prime-boost multi-antigen vaccine elicited higher frequencies of IFN-γ or TNF-α-secreting CD8^+^ and CD4^+^ T cells specific for BZLF1, EBNA1, EBNA3B, and gH/gL compared with those in mice immunized with empty DNA and wild rTTV vectors (Figure 3A-D). Of interest, in the mice that received the multi-antigen vaccine, a higher frequency of BZLF1-specific T cells was detected compared to EBNA1, EBNA3B, and gH/gL-specific T cells (Figure 3A-D). The BZLF1, EBNA1, EBNA3B, and gH/gL specific T cell responses remained at similar levels when the four antigens were administered individually or in combination (Figure 3A-D).

These data indicated that the heterologous prime-boost multi-antigen EBV vaccine expressing BZLF1, EBNA1, EBNA3B, and gH/gL were safe and induced robust and durable antigen-specific humoral and cellular immune responses in mice.

### The heterologous prime-boost multi-antigen EBV vaccine protected humanized mice from lethal EBV challenge

We next assessed the protective efficacy of the heterologous prime-boost multi-antigen vaccine against EBV challenge *in vivo*. Because we aimed to design a vaccine to prevent the development of EBV-related B cell lymphomas. Our previous research has shown that low-dose EBV challenge in humanized mice led to persistent infection without developing lymphomas, whereas high-dose EBV infection in humanized mice induced the formation of a fatal lymphoma [37]. Therefore, we selected high-dose EBV in our experiments to assess whether the multi-antigen vaccines can confer protection against EBV infection-induced fatal B cell lymphomas in humanized mice.

To generate humanized mice for these studies, CB17.Cg-*Prkdc^scid^Lyst^bg-J^*/Crl (SCID-Beige) mice were chosen as the recipients of human lymphocyte transplantation, given that the mice lack T and B cells and have impaired NK cell function [38, 39]. Four-week-old SCID-Beige mice were irradiated with X-rays and treated with clodronate liposomes [40]. The following day, 1.5 × 10^7^ human peripheral blood mononuclear cells (hPBMCs) from healthy HLA-A*02:01^(+)^ and HLA-A*02:03^(+)^ EBV-seropositive donors (dono1 and donor 2, Table 1) were engrafted by intraperitoneal (i.p.) injection. hCD45^+^ cells had established stable engraftment in SCID-Beige mice by week four after transplantation (Figures S7A and S7B). Flow cytometry analyses showed that ∼65.7% and ∼1.5% of hCD45^+^ cells in the peripheral blood were hCD3^+^ T cells and hCD19 ^+ ^hCD20^+^ B cells, respectively (Figures S7C and S7D). We also observed the engraftment of hCD45^+^ cells, hCD3^+^ T cells, and hCD20^+^ B cells in the spleen, liver, lung, and kidney of humanized mice by immunohistochemistry (IHC) (Figure S7E). Functional human CD3 ^+ ^CD8^+^ T cells and CD3 ^+ ^CD4^+^ T cells that can be activated *ex vivo* were detected in the peripheral blood of humanized mice at week eight after transplantation (Figures S8A and S8B).

**Table 1.** The Human Leukocyte Antigen (HLA) of human PBMCs donors.

Donor	A	B	C	DPB1	DRB1	DQB1
1	A*24:02:01;A*02:01:01	B*55:02:01;B*15:12:01G	C*12:03:01;C*03:03:01	DPB1*05:01:01;DPB1*02:01:02	DRB1*14:05:01;DRB1*12:02:01	DQB1*03:01:01;DQB1*05:03:01
2	A*02:03:01G;A*02:03:01G	B*38:02:01G;B*15:02:01	C*07:02:01;C*08:01:01	DPB1*05:01:01;DPB1*21:01:01G	DRB1*16:02:01;DRB1*12:02:01	DQB1*03:01:01;DQB1*05:02:01
3	A*24:02:01;A*02:03:01G	B*52:01:01;B*40:01:02	C*12:02:02;C*03:17:01	DPB1*14:01:01;DPB1*04:01:01	DRB1*16:02:01;DRB1*14:04:01G	DQB1*05:03:01;DQB1*05:02:01
4	A*02:07:01;A*30:01:01	B*40:01:02;B*37:01:01	C*07:02:01;C*06:02:01	DPB1*05:01:01;DPB1*05:01:01	DRB1*10:01:01;DRB1*08:03:02	DQB1*06:01:01;DQB1*05:01:01

**Figure 4.**
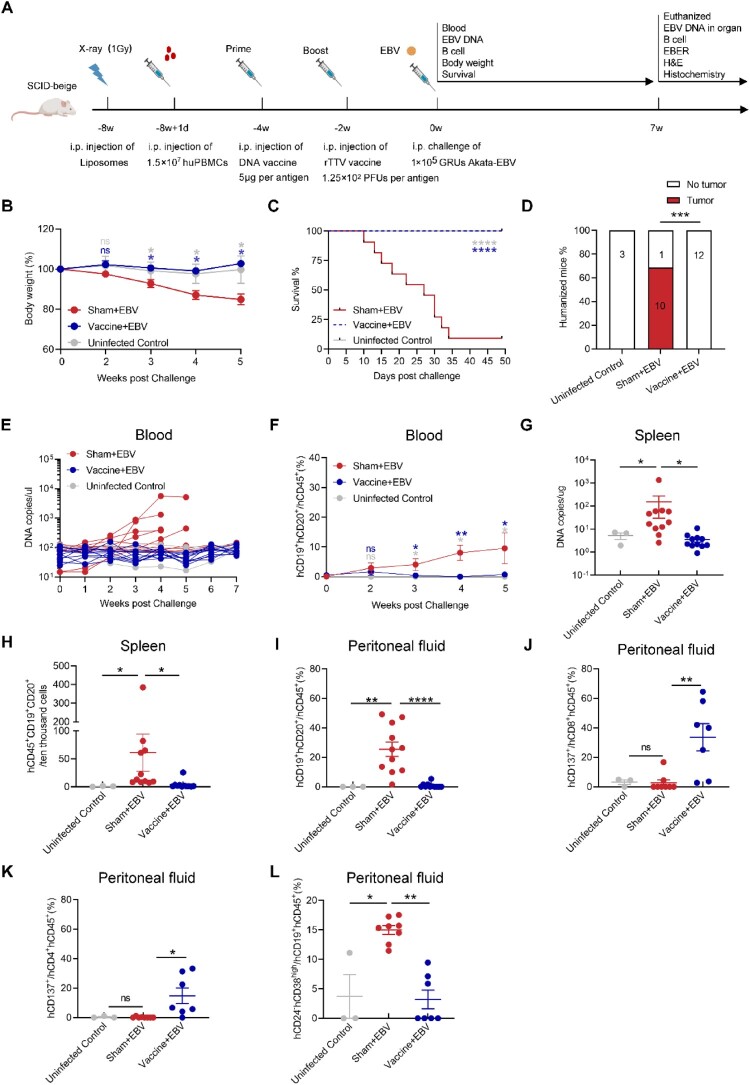
Vaccination of humanized mice with the multi-antigen DNA and the multi-antigen rTTV vaccine confers protective immunity. (A) Experimental timeline. The figure was created from Biorender.com. Humanized mice were vaccinated with DNA vaccine (5 μg per antigen) and rTTV vaccine (1.25 × 10^2^ PFUs per antigen) via intraperitoneal (i.p.) injection and heterologous prime-boost immunization schedules with a two-week interval. Two weeks after the final immunization, humanized mice were bled and challenged with Akata EBV equivalent to 1 × 10^5^ Raji infectious units. Uninfected Control mice receive no vaccine and no virus, sham + EBV mice receive empty vector and virus, and vaccine + EBV receive vaccine and virus. Body weight (B), survival (C), tumour incidence (D), EBV copies (E), and hCD19 ^+ ^hCD45^+^ and hCD20^+^ hCD45^+^ B cells (F) in the peripheral blood of humanized mice were monitored during the experiment. Each line in (E) represents an individual mouse. The DNA copy numbers in the spleen at the end of the experiment (G). The number of hCD45^+^ hCD19^+^ and hCD45 ^+ ^hCD20^+^ B cells/10^4^ cells in the spleen (H) and the percent of hCD19 ^+ ^hCD20^+^ B cells in the peritoneal fluid (I) at the end of the experiment. (J-L) At necropsy, the frequency of (J) hCD8 ^+ ^hCD137^+^, (K) hCD4 ^+ ^hCD137^+^, and (L) hCD19 ^+ ^hCD24^-^hCD38^high^ cells were measured in peritoneal fluid. Data schematics for uninfected control, sham + EBV, and vaccine + EBV are coloured gray, blue, and red, respectively. Each dot in (E and G-L) represents an individual mouse. Uninfected Control n = 3, Sham + EBV n = 11, and Vaccine + EBV n = 11 in B-I. Uninfected Control n = 3, Sham + EBV n = 8, and Vaccine + EBV n = 7 in J-L. Survival curves were compared with the log-rank test. Tumour incidences (%) were compared with the two-tailed Fisher^'^s exact text. The data are shown as the mean ± standard error of the mean (SEM). Other statistical analyses were performed using Mann-Whitney rank-sum test (ns, *P*≥ 0.05; *P < 0.05; **P < 0.01; ***P < 0.001; ****P < 0.0001).

As shown in Figure 4A, humanized mice were primed with the empty DNA vaccine (20 μg) or the multi-antigen DNA vaccine (5 μg per antigen) injected intraperitoneally by polyplus *in vivo*-jetPEI™ transfer reagent. Two weeks later, mice were boosted with the wild-type rTTV virus (5 × 10^2^ PFUs) or the multi-antigen rTTV vaccine (1.25 × 10^2^ PFUs per antigen). Animals were challenged with 1 × 10^5^ Green Raji Units (GRUs) of Akata EBV two weeks after the boost immunization. We also included an uninfected control group that received neither vaccine nor EBV challenge. All animals were monitored for body weight, survival, and the virological and immunological parameters up to seven weeks after the EBV challenge.

All animals treated with the heterologous prime-boost multi-antigen vaccine (the vaccine group) survived the EBV challenge and maintained relatively stable body weight without visible pathological changes and tumour formation (Figure 4B-D). By contrast, the animals immunized with the heterologous prime-boost empty vaccine (the sham group) began significantly losing body weight from the three-week post-EBV challenge (Figure 4B). Only one out of 11 mice in the sham group survived at week seven after the EBV challenge (Figure 4C). EBV DNA copy numbers in blood from the mice treated with the heterologous prime-boost empty vaccine rapidly increased in the peripheral blood from week two after the challenge, and six out of 11 mice developed EBV viremia (Figure 4E). By contrast, the animals treated with the heterologous prime-boost multi-antigen vaccine or those in the uninfected control group remain free of viremia after the challenge (Figure 4E).

EBV viremia in the blood is closely related to EBV-induced lymphoproliferative disorder (EBV-LPD). In the control group without vaccination, EBV challenge caused the death of humanized mice about week two post infection. EBV DNA in blood continued to rise from week two when compared to the mice protected by vaccine without development of LPD. These results indicate that lymphoma typically appears about two weeks post EBV challenge in humanized mice without vaccination. We observed that the percentage of hCD19 ^+ ^hCD20^+^ B cells in the peripheral blood of the animals treated with the heterologous prime-boost empty vaccine significantly increased from ∼0.11% to ∼21.1% in week seven after the EBV challenge. In contrast, the percentage of hCD19 ^+ ^hCD20^+^ B cells in the peripheral blood of the animals treated with the heterologous prime-boost multi-antigen vaccine, as well as the animals in the uninfected control group, were maintained at a low level following a transient increase during two to three weeks post EBV challenge (Figure 4F).

Furthermore, EBV DNA level and human B cell infiltration in mouse spleen and peritoneal fluid collected at necropsy were evaluated. The levels of EBV DNA and the infiltration of human B cells in the spleen exhibited a similar trend to those observed in the peripheral blood. EBV DNA copy number and human B cell numbers in the spleen were significantly lower in the multi-antigen vaccine and uninfected control groups than in the empty vaccine-treated group (Figure 4G-H). We also found a significantly higher percentage of human B cells in the peritoneal fluid of mice in the empty vaccine-treated group (Figure 4I). Relative to the animals treated with the heterologous prime-boost empty vaccine, the frequency of human activated CD8^+^ T cell (hCD8 ^+ ^CD137^+^) and activated CD4^+^ T cell (hCD4 ^+ ^hCD137^+^) were lower than the animals treated with the heterologous prime-boost multi-antigen vaccine (Figure 4J and K, Figure S9). At the same time, animals that received the heterologous prime-boost empty vaccine had a higher frequency of proliferating memory B cells (hCD19 ^+ ^hCD24^-^hCD38^high^) than the uninfected control group and the vaccine group (Figure 4L, Figure S10).

In summary, these results demonstrate that the heterologous prime-boost multi-antigen vaccine can control EBV infection and confer robust protection against EBV viremia after lethal EBV challenge *in vivo*.

### The heterologous prime-boost multi-antigen EBV vaccine inhibited the formation of EBV-induced B cell lymphomas in humanized mice

**Figure 5.**
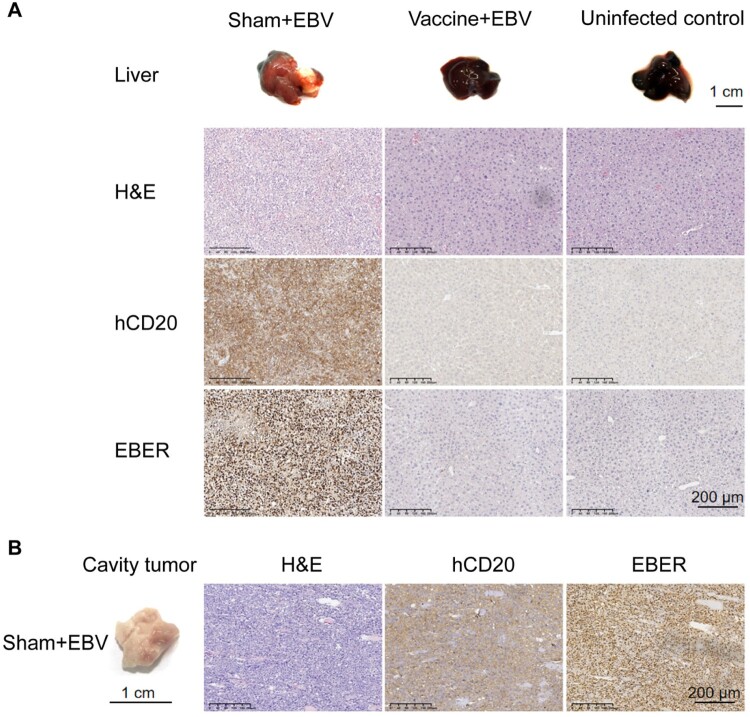
Heterologous prime-boost vaccination with multi-antigen EBV vaccine reduces EBV replication and tissue damage in humanized mice. Representative liver sections (A) and cavity tumour (B) were stained for hematoxylin and eosin (H&E), human CD20 (hCD20), and EBV-encoded RNA (EBER) at necropsy. The scale bars are indicated.

To study the impact of protection at the tissue level, we collected the animals’ lymph nodes, spleens, and livers at the autopsy. We found that 10 out of 11 humanized mice succumbing to the EBV challenge developed tumours (Figure 4D). The profound and visible changes were observed in the livers and the abdominal cavity tissue. Morphologically, mice's livers treated with the heterologous prime-boost empty vaccine were significantly enlarged, and tumours were visible (Figure 5A). By contrast, the livers from mice treated with the heterologous prime-boost multi-antigen vaccine and the uninfected control group were normal in size, without visible tumours (Figure 5A). In the sham group of mice, substantial tumours were detected in the abdominal cavity tissue. Conversely, the vaccine and the uninfected control group exhibited no tumour in the abdominal cavity tissue (Figure 5B). The *in situ hybridization* for Epstein–Barr virus-encoded RNAs (EBERs) and immunohistochemistry (IHC) staining for hCD20 confirmed the development of typical B cell lymphomas that were hCD20 ^+ ^EBER^+^ in the livers and the abdominal cavity tissue of the mice treated with the empty vaccine (Figure 5A and B). hCD20 ^+ ^EBER^+^ B cell lymphomas were abundant and widely distributed across the tissue section (Figure 5A and B). By contrast, hCD20 ^+ ^EBER^+^ cells were rarely found in the livers of the mice of the multi-antigen vaccine immunization group (Figure 5A). Meanwhile, in the spleen sections of mice treated with the empty vaccine, several hCD20 ^+ ^EBER^+^ B cell clusters were identified, while they were rare in the mice treated with the multi-antigen vaccine and in the uninfected control group (Figure S11).

These results show that the heterologous prime-boost multi-antigen vaccine can significantly reduce viral replication and prevent EBV-induced B cell lymphomas in the humanized mouse model.

### The heterologous prime-boost multi-antigen EBV vaccine elicited antigen-specific T cell responses against BZLF1, EBNA1, and EBNA3B in humanized mice

**Figure 6.**
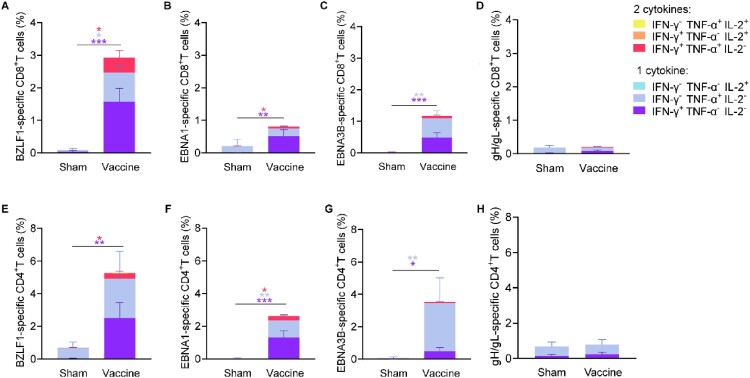
Heterologous prime-boost vaccination with multi-antigen EBV vaccine elicits robust BZLF1, EBNA1, and EBNA3B-specific T cell responses in humanized mice. Humanized mice were vaccinated with DNA vaccine (5 μg per antigen) and rTTV vaccine (1.25 × 10^2^ PFUs per antigen) via intraperitoneal (i.p.) injection and heterologous prime-boost immunization schedules with a two-week interval. The peripheral blood was harvested and stimulated with BZLF1, EBNA1, and EBNA3B peptide pools and gH/gL protein two weeks after the final immunization. Percentages of antigens-specific CD8^+^ and CD4^+^ T cells producing IFN-γ, TNF-α, and IL-2 and frequencies of combinations of cytokines produced by CD8^+^ (A-D) and CD4^+^ (E-H) T cells are shown. Sham n = 6, Vaccine n = 8. The data are shown as the mean ± standard error of the mean (SEM). *P* values were determined by Mann-Whitney rank-sum test (ns, *P*≥ 0.05; *P < 0.05; **P < 0.01; ***P < 0.001; ****P < 0.0001).

The multi-antigen EBV vaccine provided potent protection against EBV-induced lymphomas, which might correlate to its ability to elicit antigen-specific T cell responses. We next examined the T cell responses against BZLF1, EBNA1, EBNA3B, and gH/gL antigens induced by the heterogenous prime-boost multi-antigen vaccine in the humanized mice which hPBMCs from healthy HLA-A*02:03^(+)^ and HLA-A*02:07^(+)^ EBV-seropositive donors (donor 3 and donor 4, Table 1). Two weeks after the final immunization, antigen-specific T cells were assessed before the EBV challenge. Peripheral blood lymphocytes were obtained, and the ICS assay was used to assess the human CD8^+^ and CD4^+^ T cell responses specific to BZLF1, EBNA1, EBNA3B, and gH/gL antigens (Figure S12). The prime-boost multi-antigen EBV vaccine induced a significant proportion of IFN-γ-producing CD8^+^ and CD4^+^ T cells against antigens BZLF1, EBNA1, and EBNA3B (Figure 6A-C and E-G). In contrast, gH/gL-specific CD8^+^ and CD4^+^ T cells were not significantly elicited after vaccination of the prime-boost multi-antigen EBV vaccine (Figure 6D and H). Notably, the frequency of BZLF1-specific CD8^+^ T cells was higher compared to EBNA1 and EBNA3B-specific cells elicited by the multi-antigen vaccine (Figure 6A-C). Polyfunctional T cells that simultaneously produce multiple cytokines, e.g. IFN-γ, TNF-α, or IL-2, possess potent cytotoxicity against antigen-expression cells. A significant number of IFN-γ/TNF-α-producing CD8^+^ T cells against BZLF1 were detected in the vaccination group (Figure 6A). These observations in a humanized mouse model indicated a hierarchical pattern of cellular responses towards the EBV antigens, with BZLF1 eliciting dominant CD8^+^ T cell responses. The response to EBNA3B and EBNA1 was comparatively less pronounced, while the T cell responses to gH/gL were almost undetected. Proteins are usually less efficient at stimulating T cell responses. This might be the technical reason for the undetectable gH/gL-specific T cell responses.

Additionally, following reconstruction and immunization, humanized mice exhibited an absence of specific IgG and IgM for gH/gL in serum samples (Figure S13), which could be due to the lack of an effective germinal centre for the mature of antibody-producing B cells in humanized mice. These results indicate that the prime-boost multi-antigen vaccine in the humanized mouse model activated BZLF1, EBNA1, and EBNA3B-specific T cells.

### The multi-antigen and single-antigen BZLF1 vaccines provided superior protection against the lethal dose EBV challenge compared to single-antigen EBNA1 and EBNA3B vaccines

To further evaluate the contribution of each antigen to the protection efficacy of the multi-antigen vaccine, we used the humanized mouse model to compare the protection efficacy of the multi-antigen EBV vaccine expressing four antigens with the multi-antigen vaccine expressing three antigens, BZLF1, EBNA1, and EBNA3B as well as four single-antigen vaccines expressing each single-antigen BZLF1, EBNA1, EBNA3B, or gH/gL. PBMCs from healthy HLA-A*02:03^(+)^ and HLA-A*02:07^(+)^ EBV-seropositive donors (donor 3 and donor 4, Table 1). We adopted the DNA-prime and rTTV-boost vaccination strategy for all the vaccines tested for the comparison. The sham group was immunized with an empty DNA vector and wild rTTV as a negative control. Two weeks after the final vaccination, all animals were challenged with 1 × 10^5^ GRUs of Akata EBV via the intraperitoneal (i.p.) route (Figure S14).

**Figure 7.**
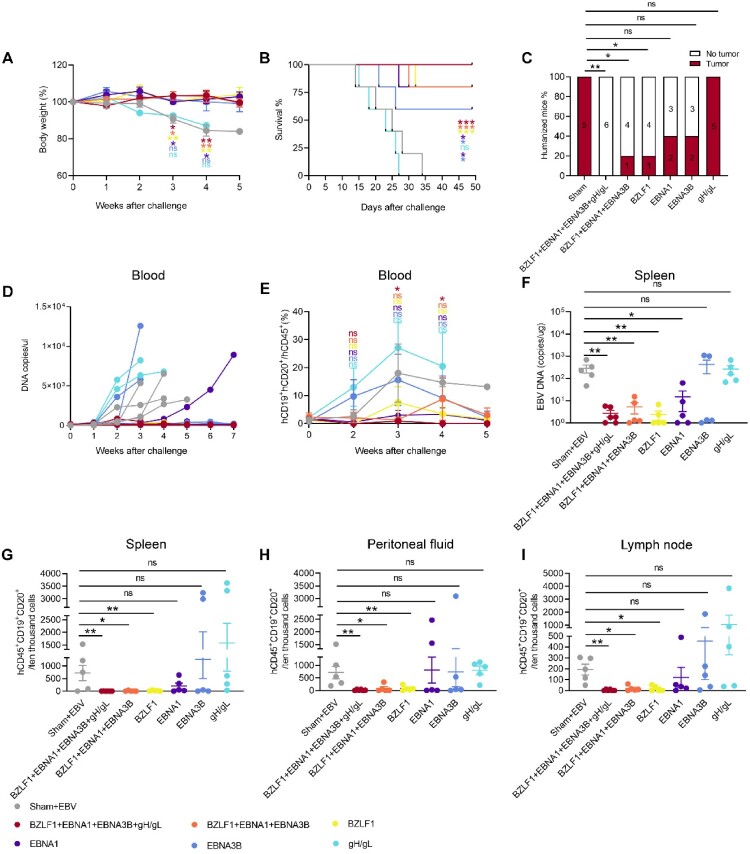
The multi-antigen EBV vaccine provides more potent protection against EBV infection than its single-antigen vaccine, except for the single-antigen BZLF1 vaccine. Body weight (A), survival (B), tumour incidence (C), EBV copies (D), and hCD19 ^+ ^CD20^+^ B cells (E) in the peripheral blood of mice were monitored during the experiment. Each line in (D) represents an individual mouse. The DNA copy numbers in the spleen at the end of experiment (F). The number of hCD45 ^+ ^CD19 ^+ ^CD20^+^ cells/10^4^ cells in the spleen (G), peritoneal fluid (H), and lymph node (I) at the end of the experiment. Each dot in (F, G, H, and I) represents an individual mouse. The multi-antigen vaccine group expressing four antigens n = 6, the multi-antigen vaccine group expressing BZLF1, EBNA1, and EBNA3B n = 5, all single-antigen vaccine group n = 5, and the sham group n = 5. Survival curves were compared with the log-rank test. Tumour incidences (%) were compared with the two-tailed Fisher's exact text. The data are shown as the mean ± standard error of the mean (SEM). Other statistical analyses were performed using Mann-Whitney rank-sum test (ns, *P*≥ 0.05; *P < 0.05; **P < 0.01; ***P < 0.001; ****P < 0.0001).

Severe diseases were observed in the sham group and the mice receiving the gH/gL monovalent vaccine. These mice experienced significant weight loss (Figure 7A) and succumbed to mortality (Figure 7B) within five weeks following the EBV challenge. All mice in the sham and single-antigen gH/gL vaccine groups developed hCD20 ^+ ^EBER^+^ B cell lymphomas, demonstrating no protection (Figure 7C). Compared to the multi-antigen vaccine groups, after immunization with the gH/gL single-antigen vaccine group, the number of EBV copies and the percentage of hCD19 ^+ ^CD20^+^ B cells in the peripheral blood rapidly increased, and there were also more EBV copies in the spleen (Figure 7D-F). In addition, in the gH/gL single-antigen vaccine group, a significant infiltration of hCD20 ^+ ^EBER^+^ positive B cells was found in the peritoneal fluid, spleen, and lymph node (Figure 7G-I) and was also observed in the liver and kidney (Figure S15). The lack of protection provided by the single-antigen gH/gL vaccine from EBV challenge in the humanized mouse model correlated with its inability to elicit antigen-specific cellular and humoral immune responses (Figure 6D and H, Figure S13).

The multi-antigen vaccine without gH/gL antigen provided significant protection against EBV-induced B lymphomas, comparable to the multi-antigen EBV vaccine expressing four antigens, which successfully prevented the development of EBV-LPD (Figure 7A-I and Figure S15). We further compared the protective efficacy of EBV-LPD by single-antigen vaccines expressing BZLF1, EBNA1, or EBNA3B. Among them, only the single-antigen BZLF1 vaccine showed a significant protective effect compared to the empty vaccine, whereas partial but non-significant protection was observed for the single-antigen EBNA1 and EBNA3B vaccines. Four out of five mice immunized with the single-antigen BZLF1 vaccine survived a seven-week follow-up (Figure 7B) and were protected from hCD20 ^+ ^EBER^+^ B lymphomas (Figure 7C and Figure S15). We found that the single-antigen BZLF1, multi-antigen vaccines are more effective in suppressing EBV replication in the peripheral blood (Figure 7D) and organs of mice, including the spleen (Figure 7F), liver (Figure S15A), and kidney (Figure S15B), compared to the single-antigen EBNA1 and EBNA3B vaccines.

Additionally, the multi-antigen and single-antigen BZLF1 vaccines were also more successful in inhibiting the proliferation of hCD19 ^+ ^CD20^+^ B cells in multiple tissues such as the peripheral blood (Figure 7E), spleen (Figure 7G), peritoneal fluid (Figure 7H), lymph node (Figure 7I), liver (Figure S15A), and kidney (Figure S15B), compared to the single-antigen EBNA1 and EBNA3B vaccines. These findings demonstrated that the single-antigen BZLF1 and multi-antigen vaccines effectively control EBV infection and prevent EBV-induced B cell proliferation in mice. The single-antigen EBNA1 and EBNA3B vaccines protected against lethal EBV challenge in three out of the five humanized mice (Figure 7C). In the single-antigen EBNA1 and EBNA3B vaccine groups, the mice that survived showed reduced EBV replication and EBV-induced B cell proliferation (Figure 7D-I and Figure S15). We found the protective effect was associated with the strength of antigen-specific T cell immunity induced by the vaccines. In the humanized mouse model, a higher level of BZLF1-specific T cell responses was observed compared to the EBNA1 and EBNA3B-specific T cell responses following administration of the multi-antigen vaccine (Figure 6A and E).

These results indicate that multi-antigen vaccines targeting latent and lytic phases of the EBV life cycle elicited broad T cell responses against BZLF1, EBNA1, and EBNA3B, but not gH/gL, which could all participate in the control of EBV-induced B cell proliferation and protection of humanized mice against lethal EBV challenge (Figure 7 and Figure S15). In addition, vaccination of humanized mice with the single-antigen BZLF1 vaccine provided significant protection against EBV infection (Figure 7 and Figure S15), indicating that BZLF1 is a promising candidate target protein in the immunosurveillance of EBV.

## Discussion

An effective EBV vaccine is urgently needed to combat EBV-associated diseases. Given the critical role and the breadth of EBV-specific T cell immune responses in the control of asymptomatic infection, it is reasonable to hypothesize that EBV vaccines capable of inducing strong virus-specific T cell responses to target both latent and lytic cycles could effectively prevent EBV-associated diseases. In this study, we used TianTan vaccinia virus as a platform to evaluate the efficacy of EBV vaccines containing BZLF1, EBNA1, EBNA3B, and gH/gL as immunogens in preventing EBV-induced B cell lymphomas in a humanized mouse model. Our findings reveal that a broad-spectrum multi-stage, multi-antigen EBV vaccine expressing all four antigens or expressing BZLF1, EBNA1, and EBNA3B provided potent protection to humanized mice against lethal EBV challenge. This protection closely correlates with the antigen-specific T cell responses elicited by the EBV vaccines. Our results highlight that the BZLF1 single-antigen vaccine induced stronger T cell responses than EBNA1 and EBNA3B single-antigen vaccines, while the gH/gL single-antigen vaccine failed to activate gH/gL-specific T cell responses. Notably, the BZLF1 single-antigen vaccine effectively protected humanized mice from EBV-induced B cell lymphomas, indicating that BZLF1 is an ideal antigen target for EBV vaccine design.

In this study, we developed a safe and immunogenic EBV vaccine, using a DNA vector and replication-competent TianTan vaccinia virus expressing viral antigens in a prime-boost strategy. Attenuated vaccinia viruses have been demonstrated to be a safe and effective vaccine platform for inducing strong and long-lasting humoral and cellular immunity in large-scale population vaccination campaigns, most notably during the global effort to eradicate smallpox [21]. Research into the vaccinia virus continued for its potential use as a vector for delivering antigens in novel vaccines for Zika [41], HIV [23], and Ebola [42]. The TianTan vaccinia virus was isolated in 1926 and attenuated through cell culture techniques in the 1960s [43]. Our study showed that the attenuated TianTan vaccinia virus is a safe vaccine platform without remnants in mice, in accordance with previous studies [30]. We demonstrated that the heterologous DNA prime-vaccinia virus boost vaccine regimen induced robust and durable humoral and cellular immune responses against BZLF1, EBNA1, EBNA3B, and gH/gL in BALB/c mice. Notably, in a humanized mouse model, the administration of only 5 × 10^2^ PFUs of the vaccinia-based vaccine was able to activate T cells, providing complete protection to the mice from B lymphomas occurrence during seven weeks following the EBV challenge. This finding highlights the efficacy of low-dose vaccinia virus-based vaccines in eliciting long-lasting protective T cell responses, consistent with previous reports [25, 44]. In clinical trials, vaccinia virus has been used to express gp350, EBNA1, and LMP2 to prevent EBV infection [45] or the treatment of NPC [46], indicating that it is a safe and effective platform for antigen delivery and worth further exploration.

**Table 2.** Primer sequences of qRT-PCR.

Gene name	Forward primer	Reverse primer
BZLF1	CTGGTGTCCGGGGGATAAT	TCCGCAGGTGGCTGCT
EBNA1	GTTCCTCGCCTTAGGTTGTA	AGCTCTCCTGGCTAGGAGTC
EBNA3B	GGATCGTCACCACCATTGT	GGTGGGATCTGAGCCTATTT
BKRF2 (gL)	TCTCCATCCTGAAGCGAAGC	TGGCACCAAACAGGTCTTCC
BXLF2 (gH)	CCAGCACCACCTATCTCAGC	CAGGATTTCTGCGTCCTGGT
GAPDH	GCACCGTCAAGGCTGAGAAC	TGGTGAAGACGCCAGTGGA

Primer sequences of qRT-PCR (5′−3′).

Multiple EBV antigens have been evaluated as potential targets for designing vaccines or therapy to prevent or treat EBV-induced malignancies, both in the humanized mouse model and clinical trials. The primary focus has been on latent antigens EBNA1, LMP1, and LMP2A to address EBV-induced B cell lymphomas [47], MS [48], or NPC [46], all of which involve latent EBV infection. Our findings show that the multivalent vaccines expressing BZLF1, EBNA1, and EBNA3B, targeting both latent and lytic stages of the EBV life cycle, provided more effective protection against the development of EBV-induced B cell lymphomas compared to the monovalent vaccines targeting only latent antigens EBNA1 or EBNA3B. A previous study has demonstrated the efficacy of a multivalent EBV VLP vaccine, comprising structural proteins (*i.e.* lytic envelope, tegument, and capsid) along with the latent antigen EBNA1, in providing potent protection against EBV infection in humanized mice. The activation of EBNA1-specific T cells played a crucial role in this protection [49]. Furthermore, the adoptive transfer of CD8^+^ T cell clones specific to EBV lytic antigen BMLF1 as well as latent antigen LMP2 has been shown to inhibit the development of B-cell lymphoma caused by EBV infection in humanized mice [50, 51]. Together with these studies, our findings support the development of EBV vaccines that elicit broad T cell responses targeting both latent and lytic stages of the EBV life cycle. Humanized mice that received the multi-antigen vaccine expressing BZLF1, EBNA1, EBNA3B, and gH/gL effectively inhibited EBV infection, reduced EBV replication, and completely prevented the development of EBV-induced B cell lymphomas.

Furthermore, in this study, the results indicate that BZLF1 is the major immunogen that offers protection against EBV challenge in our humanized mice model, whereas EBNA1 and EBNA3B play less pronounced roles and gH/gL minimal roles in protection conferred by T cell responses in humanized mice engrafted with PBMCs from HLA-A*02:03^(+)^ and HLA-A*02:07^(+)^ EBV-seropositive donors. Previous studies have reported the protective effect of BZLF1 in the control of spontaneous EBV-induced LPD [12, 14]. Our study further shows that BZLF1 is able to elicit more potent protective T cell response against LPD induced by lethal EBV challenge than the commonly tested antigens EBNA1 and EBNA3B. Although EBNA1 and EBNA3B offer partial protection, we observed that the multi-antigen vaccine induced significant T cell response against EBNA1 and EBNA3B in humanized mice. Including EBNA1 and EBNA3B immunogens with BZLF1 would elicit broader T cell responses against EBV-induced B cell transformation and proliferation.

The humanized mice generate poor antibody responses to antigen stimulation, as there are no effective geminal centres for antibody production. Therefore, this animal model is unsuitable for evaluating the protection correlated with humoral responses elicited by EBV vaccines. Although gH/gL did not exhibit strong protective T cell responses in the humanized mice in our study, it is still a promising humoral immunodominant antigen for the design of prophylactic EBV vaccine. We and others’ previous studies have shown that serum from gH/gL-immunized mice protects humanized mice against lethal EBV challenge [2, 52, 53]. In this study, we also discovered that serum from mice immunized with multi-antigen vaccine and single-gH/gL vaccine could inhibit EBV infect B cells (Figure 2J) and epithelial cells (Figure 2K) *in vitro*. Because our work focused on the protective effect of antigenic T cells, we did not further investigate the passive protective effect of serum vaccinated with multi-antigen vaccine and single-gH/gL vaccine in humanized mice. The antigen selection for the development of therapeutic EBV vaccines against LPD needs further investigation.

In our humanized model, it usually takes 15 million hPBMCs to construct a humanized mouse, the number of cells derived from each donor limited the number of humanized mice constructed. To be strict during the experiments, the humanized mice from each donor were divided to all the experimental groups to control for the donor-to-donor variation. Therefore, there were five or six humanized mice in each group, as shown in Figure 7. With the small number of mice, we may not be able to observe the statistically significant difference when the true difference in the protective effects is small between groups.

In summary, our study demonstrates that by targeting immunodominant antigens across multiple stages of the EBV life cycle, the prime-boost heterologous multi-antigen vaccine elicited a broad spectrum of CD8^+^ and CD4^+^ T cell responses against EBV BZLF1, EBNA1, and EBNA3B. These responses effectively inhibited EBV replication and protected against EBV-induced B cell lymphomas in the humanized mouse model engrafted with PBMCs from an HLA-A*02:01^(+)^, HLA-A*02:03^(+)^, and HLA-A*02:07^(+)^ individuals. Further investigation into the EBV vaccine strategy with the combination of lytic and latent antigens is warranted.

## Supplementary Material

EMI_supplement_text_final-clean.docx
