# Supplementary material for: TianTan vaccinia virus-based EBV vaccines targeting both latent and lytic antigens elicits potent immunity against lethal EBV challenge in humanized mice
Source: Emerg Microbes Infect. 2024 Oct 10;13(1):2412640. doi: 10.1080/22221751.2024.2412640 (PMC11485817; doi:10.1080/22221751.2024.2412640)
Supplement: EMI_supplement_text_final-clean.docx [file TEMI_A_2412640_SM0296.docx]

**Figure S and figure S legends**


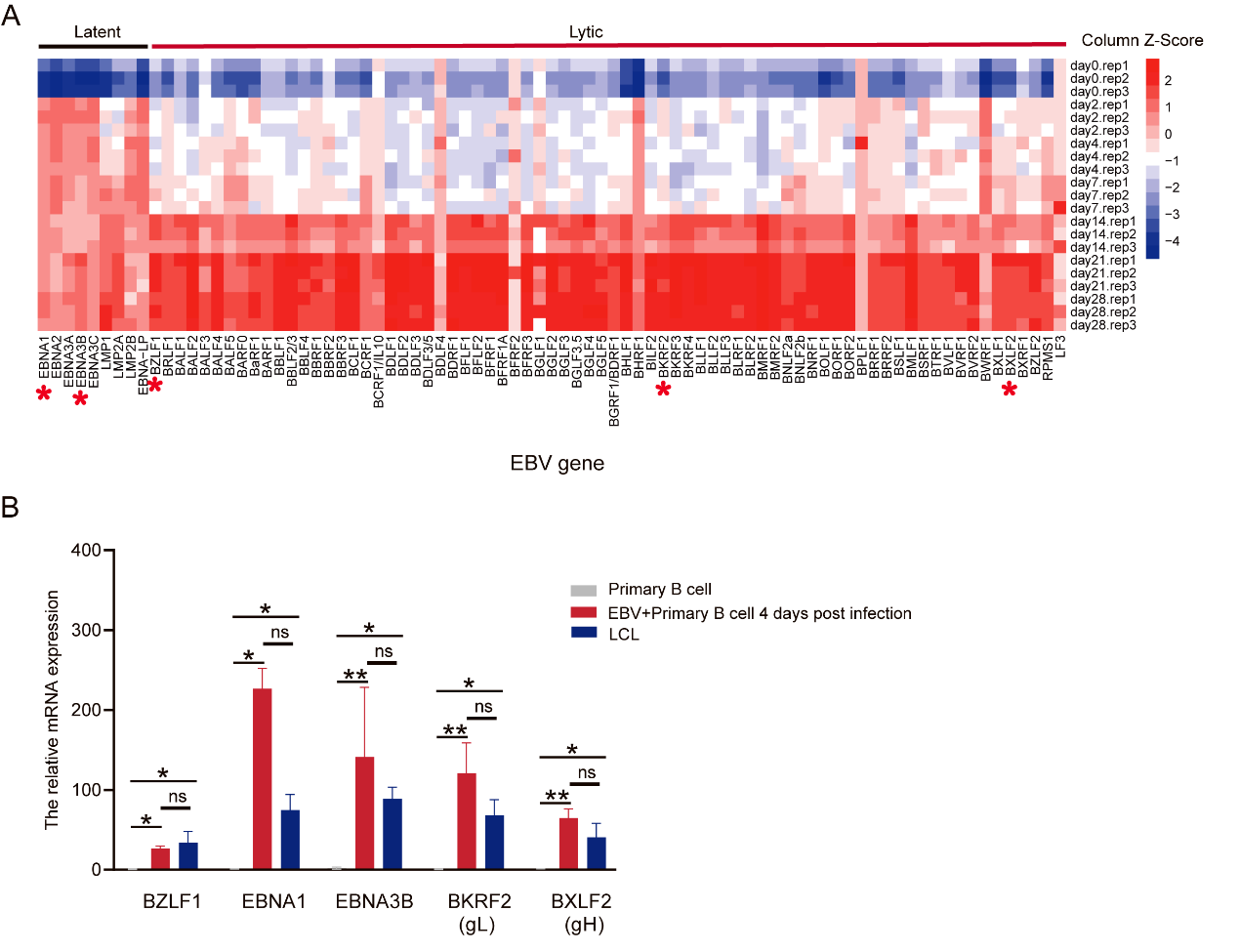
 Figure S1. EBV gene expression after primary B cell infection. (A) RNA-seq reads were mapped to the EBV genome. Normalized EBV gene expression levels are shown. (B) The relative mRNA of BZLF1, EBNA1, EBNA3B, BKRF2 (gL), and BXLF2 (gH) were calculated and normalized to the value obtained Primary B cell, EBV+primary B cell 4 days post infection, and lymphoblastoid cell line (LCL), n=3, The red star in A represents BZLF1, EBNA1, EBNA3B, BKRF2 (gL), and BXLF2 (gH). The data are shown as the mean ± standard error of the mean (SEM). Statistical analyses were performed using paired t test (ns, *P*≥ 0.05; **P*<0.05; ***P*<0.01; ****P*<0.001; *****P*<0.0001).


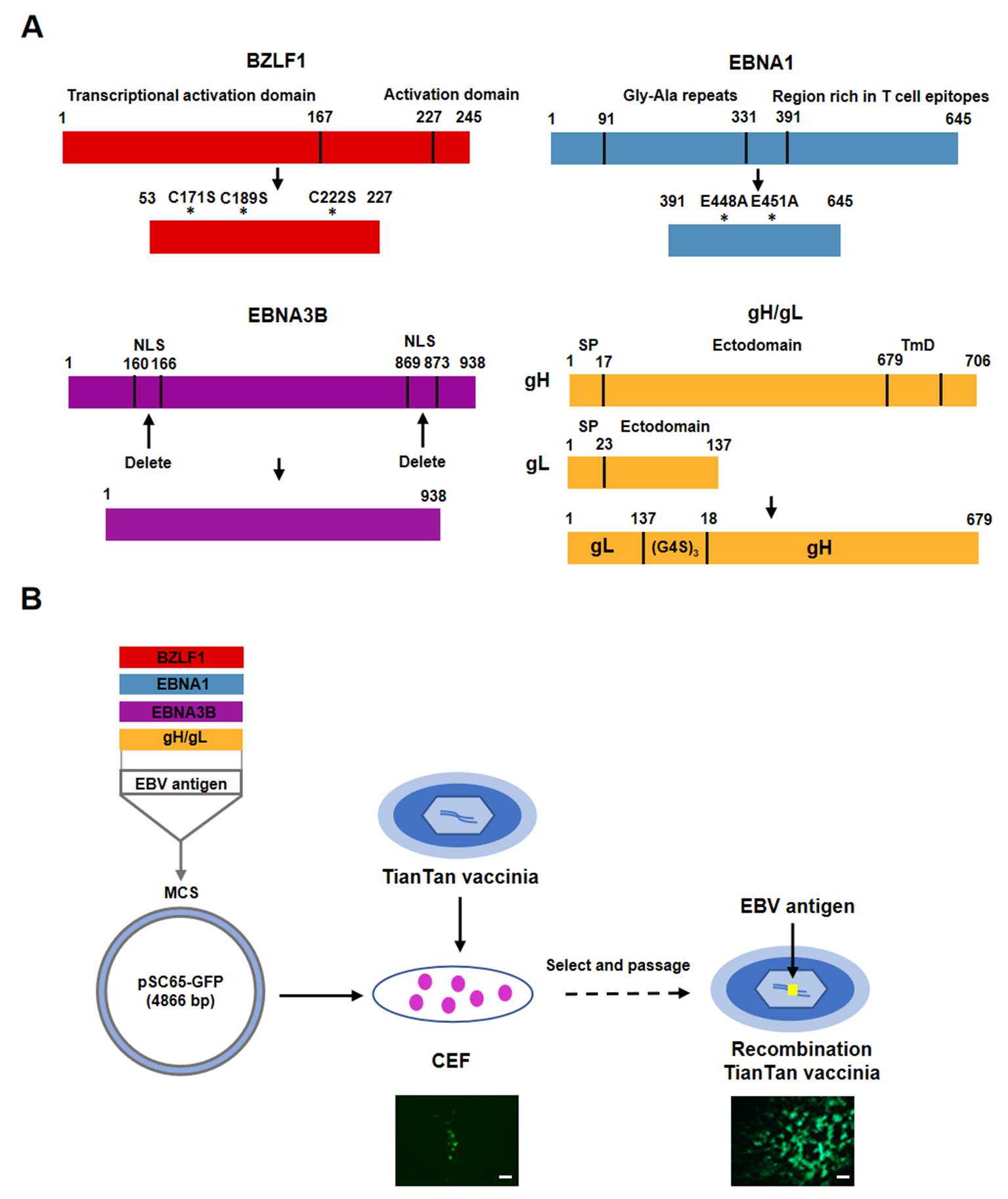


**Figure S2.** Antigen modifications and the workflow of the recombinant rTTV vaccines construction

(A) Consensus antigens were modified to avoid potentially oncogenic properties and repetitive sequences. (B) The schematic representation of the recombinant rTTV vaccines production workflow. Scar bar=20 μm.


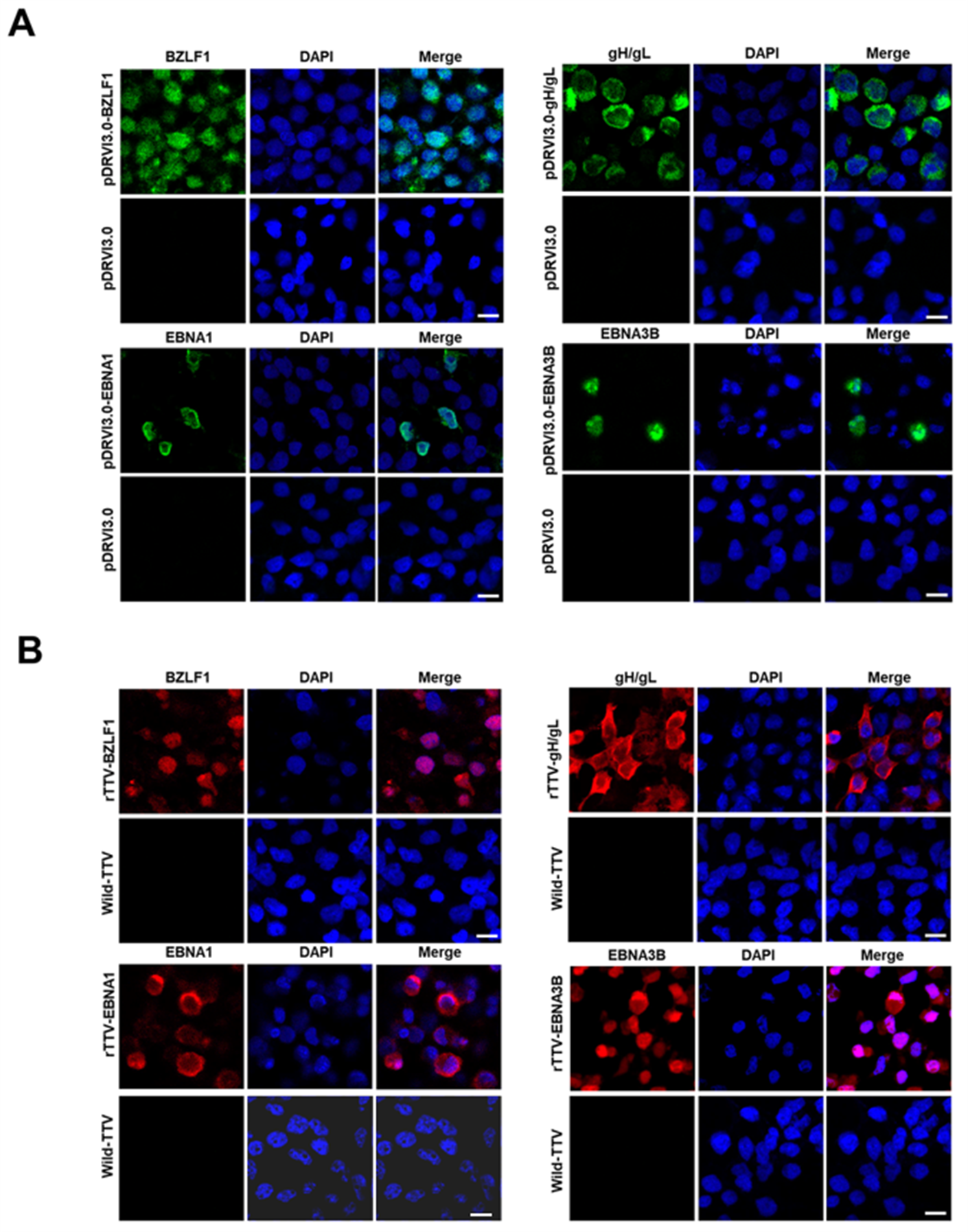


**Figure S3.** Immunofluorescence analysis of BZLF1, EBNA1, EBNA3B, and gH/gL proteins produced in recombinant DNA vaccine-transfected HEK-293T cells and recombinant rTTV vaccine-infected BHK-21 cells

(A) Immunofluorescence analysis of BZLF1, EBNA1, EBNA3B, and gH/gL proteins produced in HEK-293T cells transfected with recombinant DNA vaccine. Subconfluent HEK-293T cells infected with recombinant DNA vaccine were fixed at 48 hours post-infection, labelled with the corresponding primary antibodies, followed by the appropriate fluorescent secondary antibodies and DAPI (4',6-diamidino-2-phenylindole) to detect DNA and visualized by confocal microscopy. (B) Immunofluorescence analysis of BZLF1, EBNA1, EBNA3B, and gH/gL proteins produced in BHK-21 cells infected with recombinant rTTV vaccine. Subconfluent BHK-21 cells infected with recombinant rTTV vaccine were fixed at 24 hours post-infection, labelled with the corresponding primary antibodies, followed by the appropriate fluorescent secondary antibodies and DAPI to detect DNA, and visualized by confocal microscopy. Scar bar=10 μm.


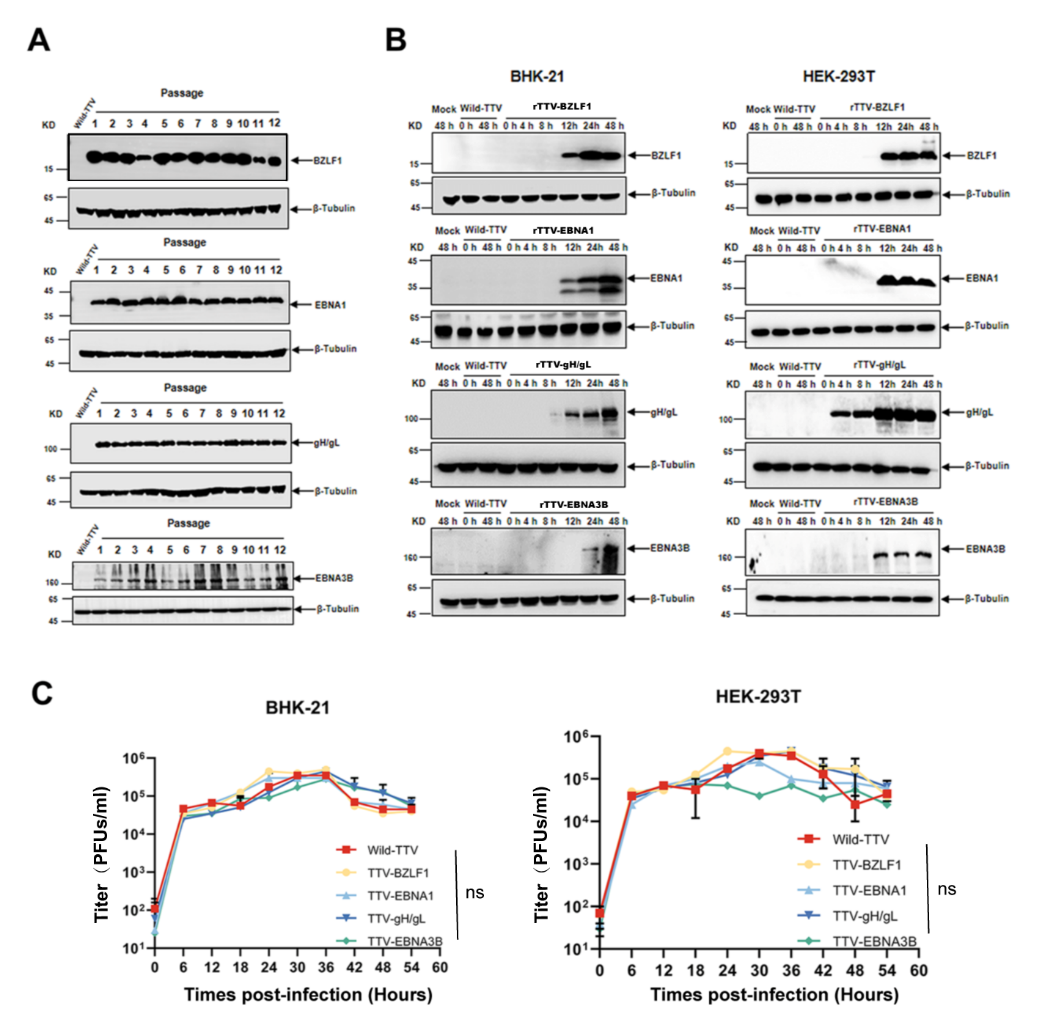


**Figure S4.** Expression kinetics, stability, and virus growth of recombinant rTTV-BZLF1, EBNA1, EBNA3B, and gH/gL

(A) The genetic stability of recombinant rTTV-BZLF1, EBNA1, EBNA3B, and gH/gL. Monolayers of BHK-21 cells were infected virus stocks from different passages at 0.1 PFU/cell with the recombinant rTTV vaccine. At 48 hours post-infection, cells were collected and detected by Western blotting. (B) Time-course expression of BZLF1, EBNA1, EBNA3B, and gH/gL proteins in BHK-21 and HEK-293T cells. The expression of BZLF1, EBNA1, EBNA3B, and gH/gL proteins at the indicated times post-infection was visualized by Western blotting in samples of mock-infected or infected cells with wild rTTV or recombinant rTTV-BZLF1, EBNA1, EBNA3B, and gH/gL. The growth of wild-TTV, rTTV-BZLF1, rTTV-EBNA1, rTTV-EBNA3B, and rTTV-gH/gL were analyzed in BHK-21 cells (C) and HEK-293T cells. The data are shown as the mean ± standard error of the mean (SEM). (ns, *P*≥ 0.05; **P*<0.05; ***P*<0.01; ****P*<0.001; *****P*<0.0001).


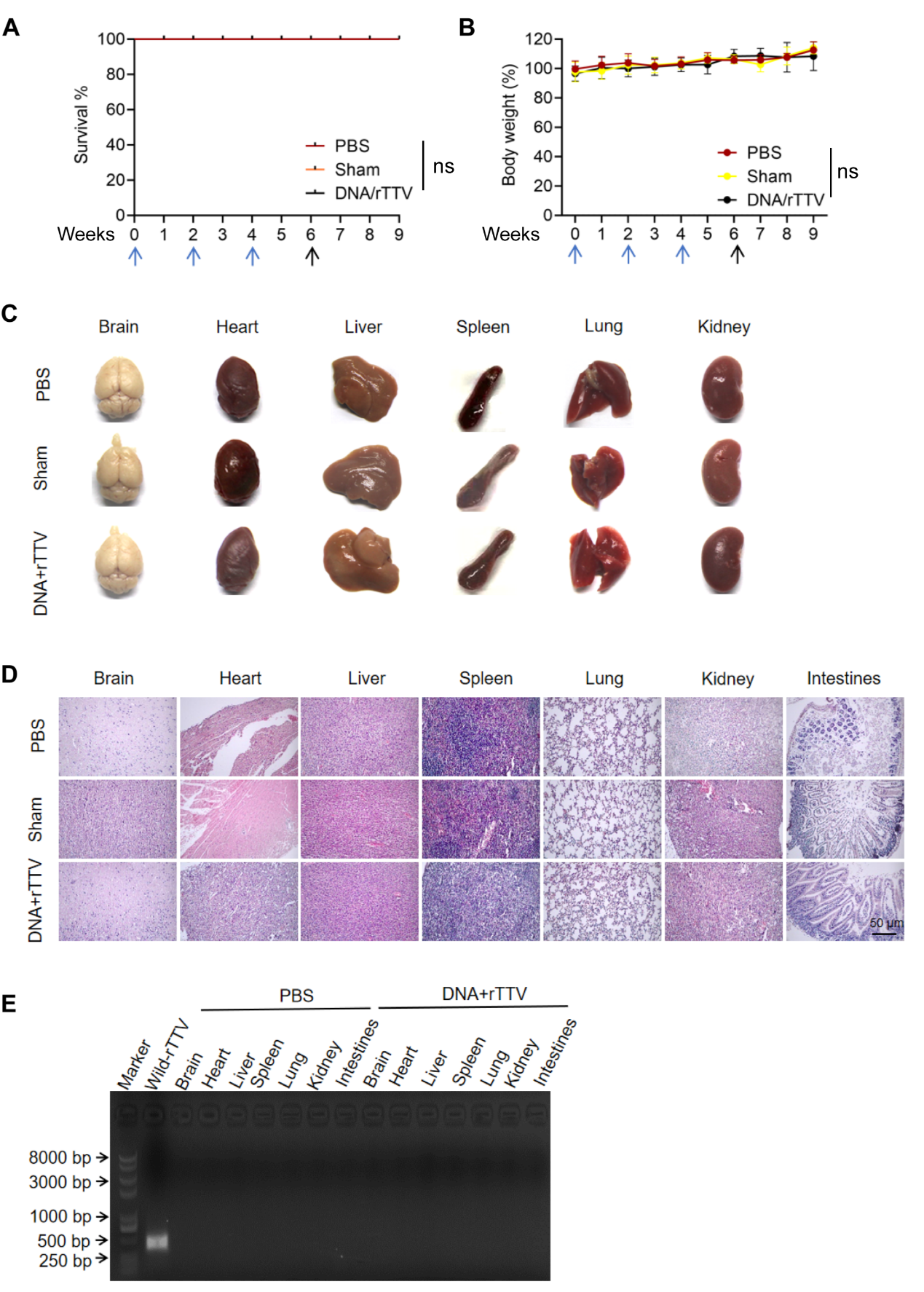


**Figure S5.** Analysis of the heterologous prime-boost multi-antigen EBV vaccine safety in BALB/c mice

(A) The survival of the BABL/c mice treated with the heterologous prime-boost multi-antigen EBV vaccine. Blue and black arrows indicate times of DNA vaccine and rTTV vaccine immunization. (B) The body weight of the BABL/c mice treated with the heterologous prime-boost multi-antigen EBV vaccine. Blue and black arrows indicate times of DNA vaccine and rTTV vaccine immunization. (C) The gross organ anatomy of the BABL/c mice treated with the heterologous prime-boost multi-antigen EBV vaccines. (D) The heart, liver, spleen, lung, brain, intestine, and kidney tissues were stained with hematoxylin and eosin (H&E) at necropsy three weeks after the final immunization (Scale bar=50 μm). (E) PCR testing showed no rTTV virus DNA residue in the heart, liver, spleen, lung, brain, intestine, and kidney tissues. Survival curves were compared with the log-rank test. Body weight statistical analysis was performed using Mann-Whitney rank-sum test. The data are shown as the mean ± standard error of the mean (SEM). (ns, *P*≥ 0.05; **P*<0.05; ***P*<0.01; ****P*<0.001; *****P*<0.0001).


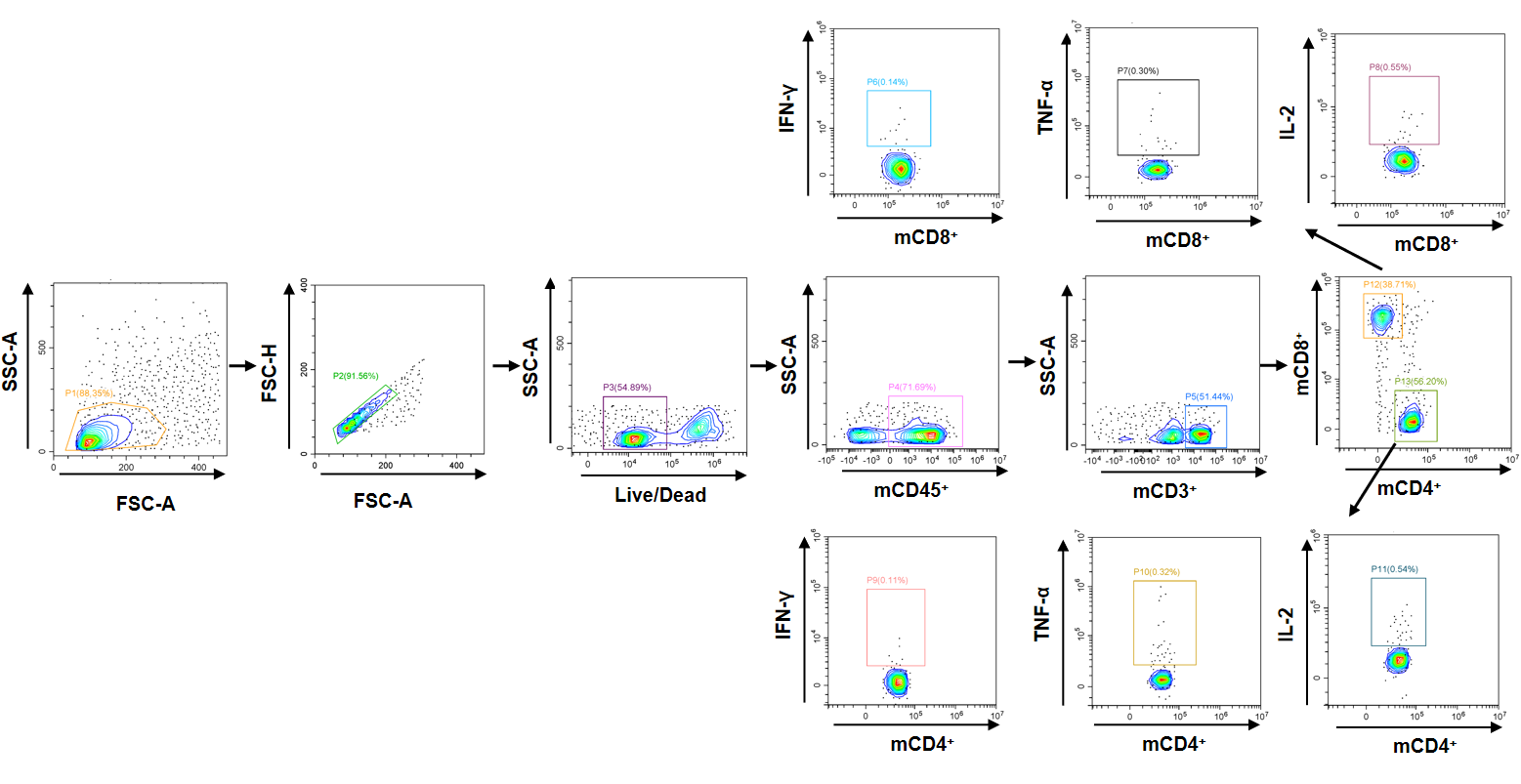


**Figure S6.** Flow cytometry gating strategy in analyzing antigen-specific T cells immune response in Figure 3.


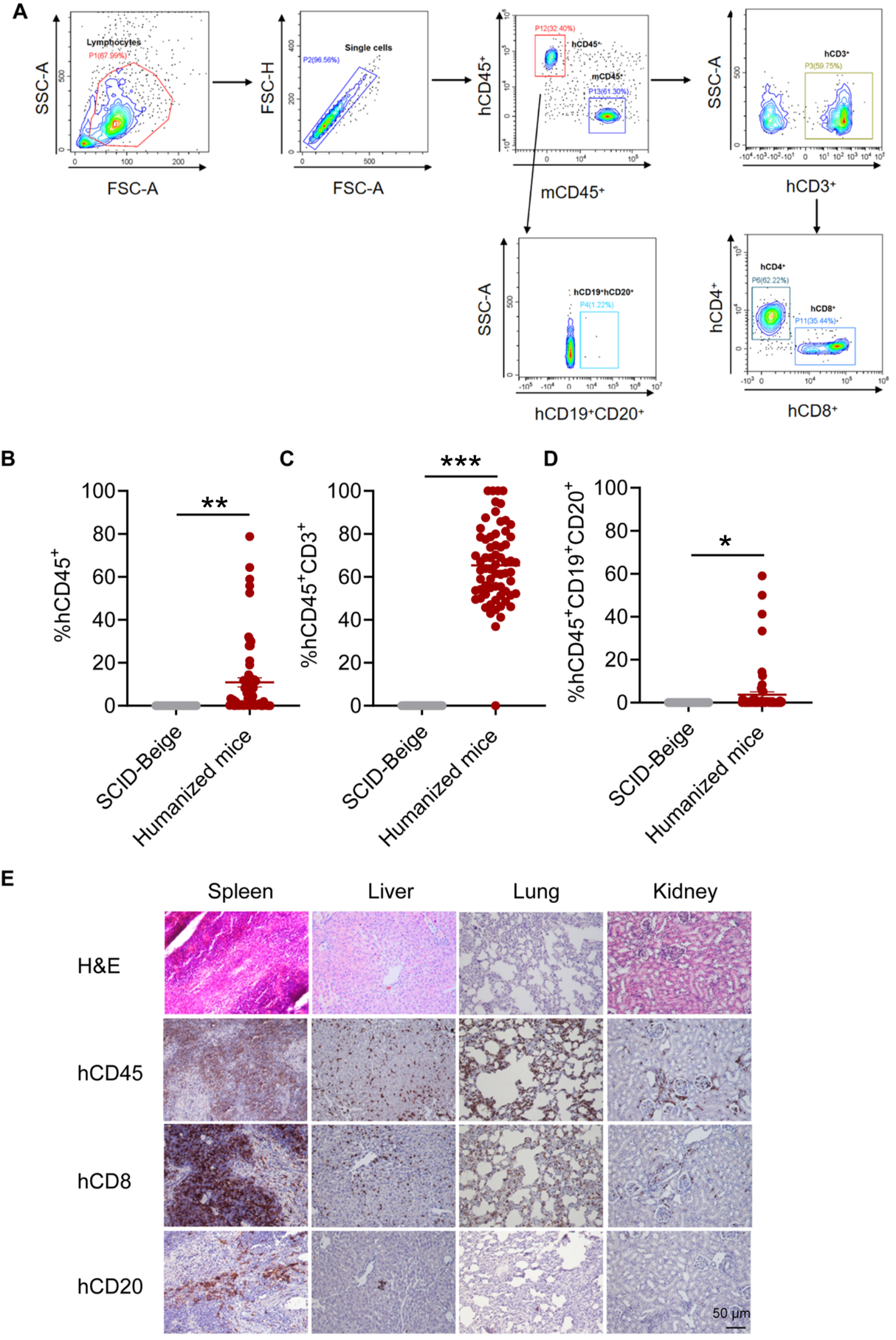


**Figure S7.** Representative flow cytometry plots showing the gating strategy used to analyze human immune cells in PBMCs collected from humanized mice (A) and the colonization of human lymphocytes in peripheral blood (B, C, and D) and various visceral tissues (E). Each dot in (B, C, and D) represents an individual mouse. The data are shown as the mean ± standard error of the mean (SEM). SCID-Beige mice n=66, Humanized mice n=66. *P*-values were determined by Mann-Whitney rank-sum test (ns, *P*≥0.05; **P*<0.05; ***P*<0.01; ****P*<0.001; *****P*<0.0001). Scale bar=50 μm.
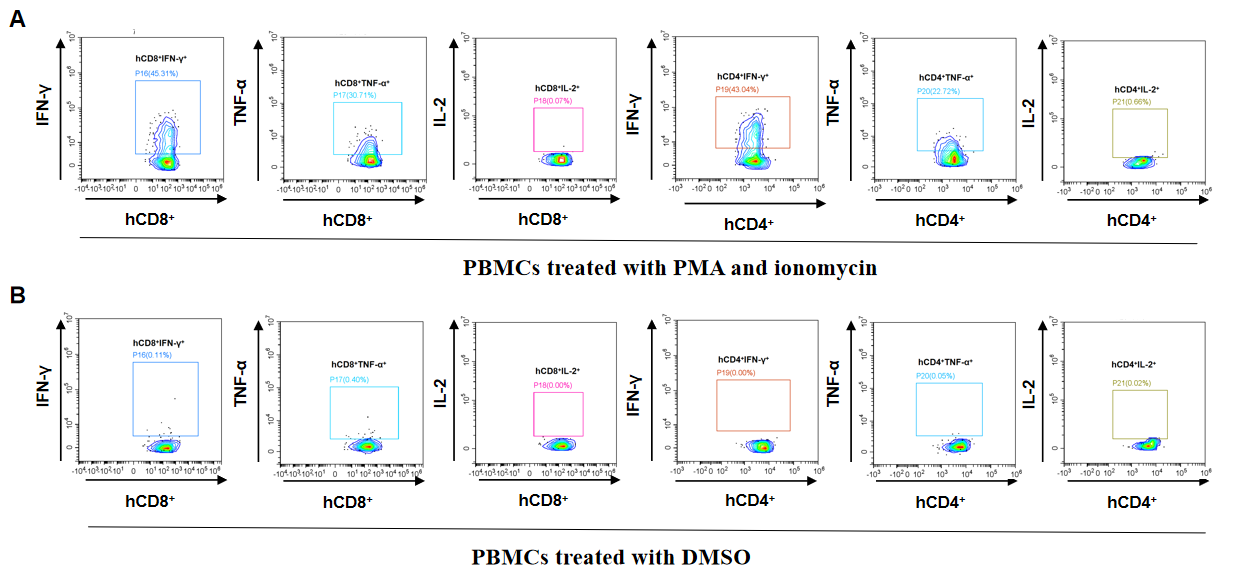


**Figure S8.** SCID-Beige humanized mice reconstructed eight weeks still have functional T cells. Human CD3^+^CD4^+^ T and CD3^+^CD8^+^ T cells in SCID-Beige humanized mice were treated with PMA, and ionomycin (A) can be activated in vitro eight weeks after reconstruction, with DMSO treated as a control (B).


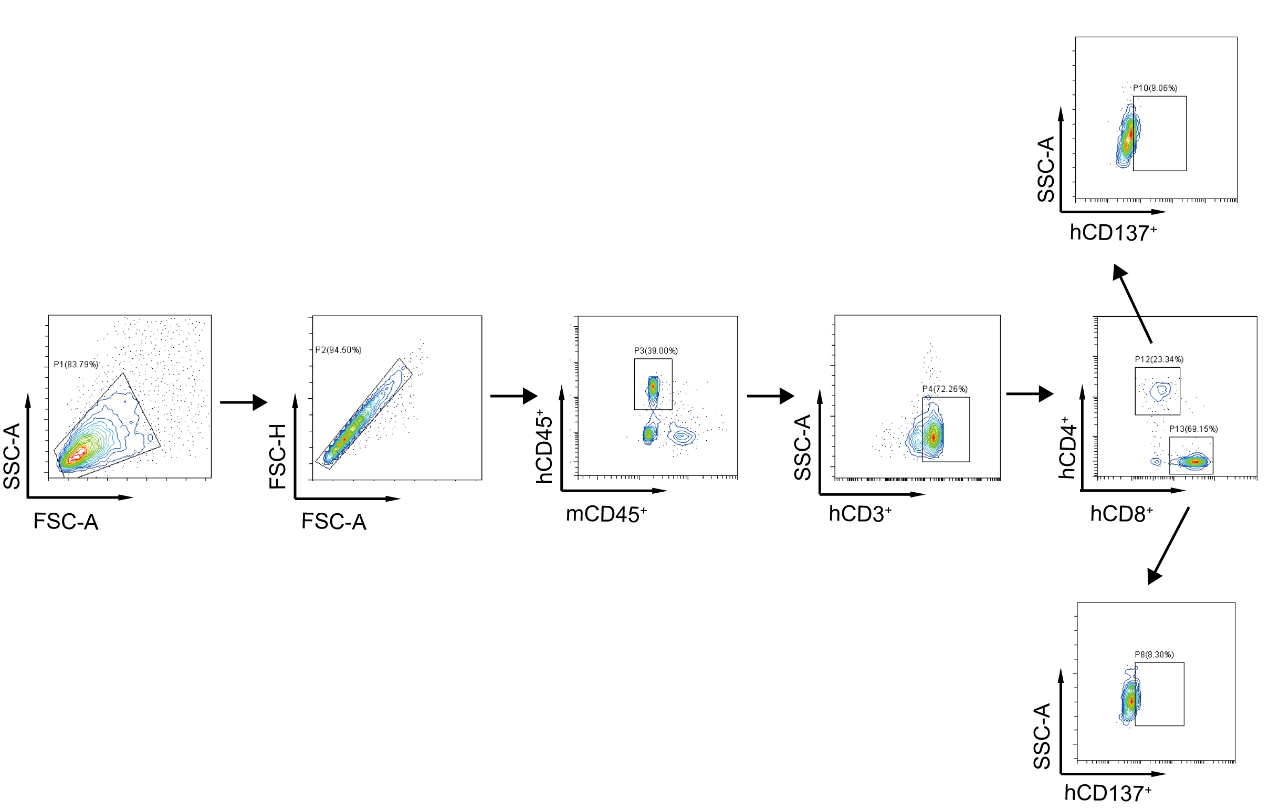


**Figure S9.** Representative flow cytometry plots showing the gating strategy used to define activated human T cell immune response in humanized mice in Figure 4.


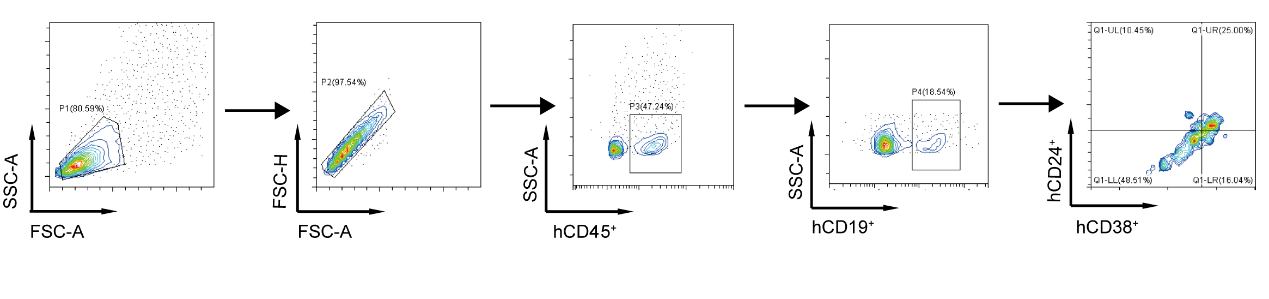


**Figure S10.** Representative flow cytometry plots showing the gating strategy used to define hCD19^+^hCD24^-^hCD38^high^ in humanized mice in Figure 4.


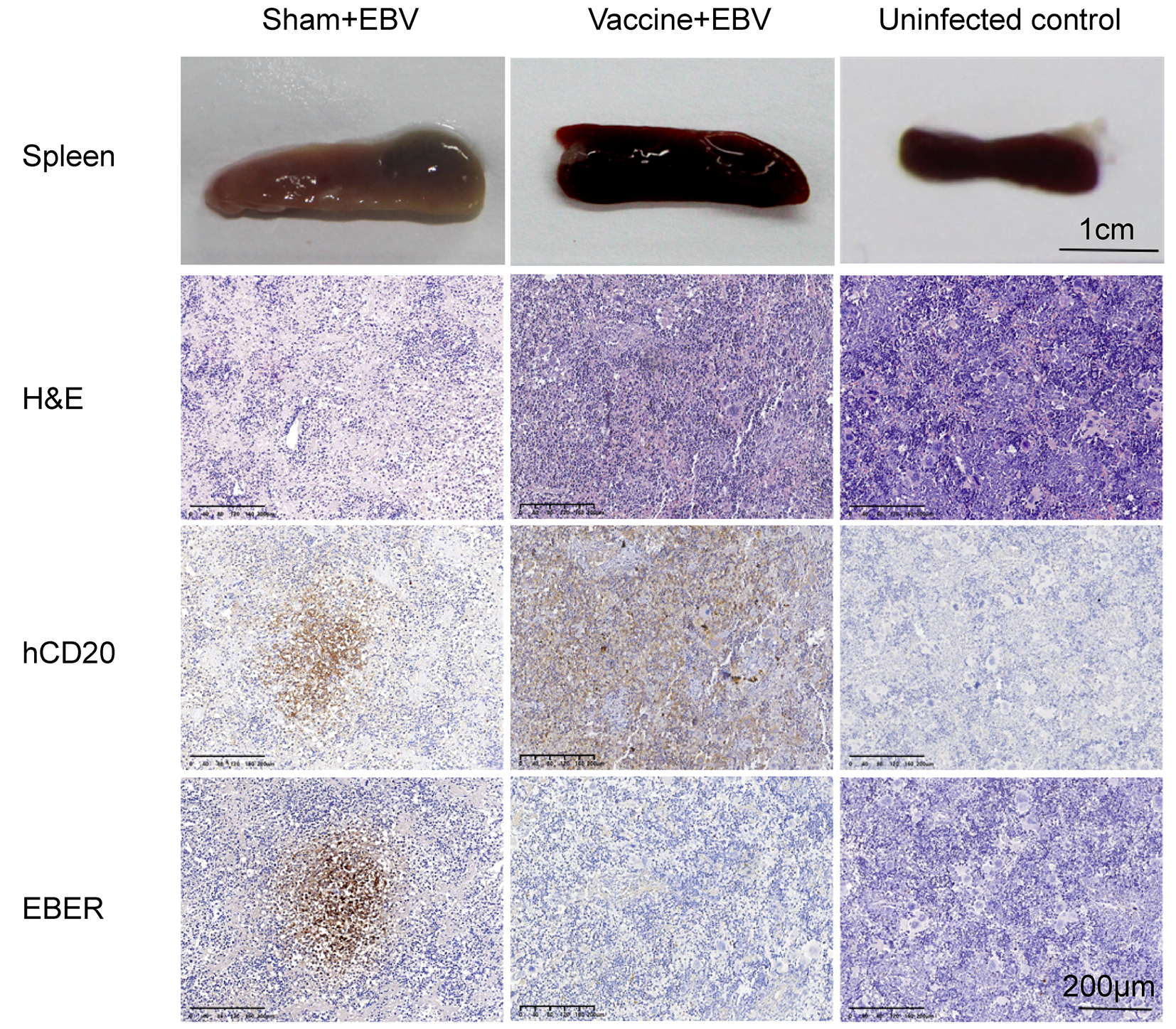


**Figure S11**. The multi-antigen DNA and multi-antigen rTTV vaccines reduce EBV replication in humanized mice. Representative of macroscopic spleens sections stained for hematoxylin and eosin (H&E), human CD20 (hCD20), and EBV encoded RNA (EBER) at necropsy. Scale bar = 200 μm.


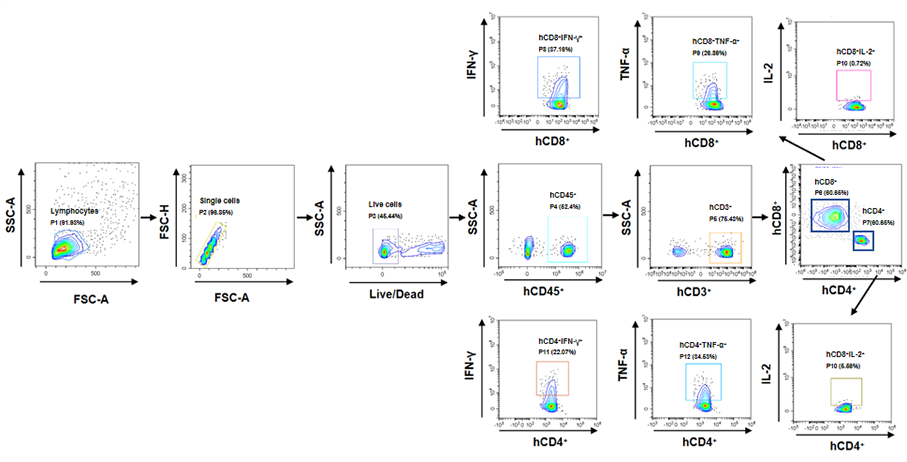
**Figure S12.** Representative flow cytometry plots showing the gating strategy used to analyze antigen-specific human T cell immune response in humanized mice.

**
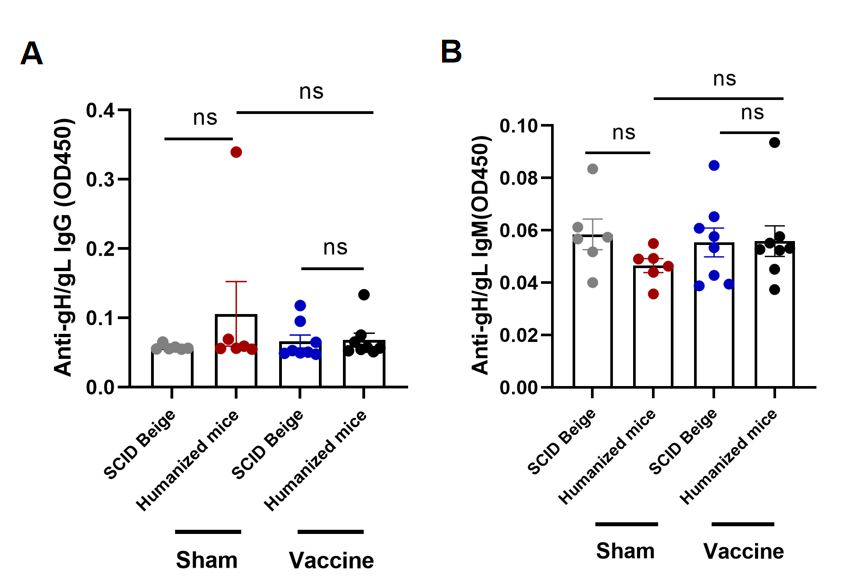
**

**Figure S13.** Heterologous prime-boost vaccination with multi-antigen EBV vaccine didn’t elicit gH/gL-specific IgG and IgM in humanized mice

Humanized mice were vaccinated with the multi-antigen DNA vaccine (5 μg per antigen) and the multi-antigen rTTV vaccine (1.25×10^2^ GFUs per antigen) via intraperitoneal (i.p.) injection and heterologous prime-boost immunization schedules with a two-week interval. The sham group mice received empty DNA and wild rTTV. The titers of anti-gH/gL IgG (A) and IgM (B) in serum were collected from SCID-beige mice and humanized mice two weeks after the final immunization. The data are shown as the mean ± standard error of the mean (SEM). The multi-antigen vaccine group n=8 and the sham group n=6. *P* values were determined by the Mann-Whitney rank-sum test (ns, *P*≥ 0.05; **P*<0.05; ***P*<0.01; ****P*<0.001; *****P*<0.0001).


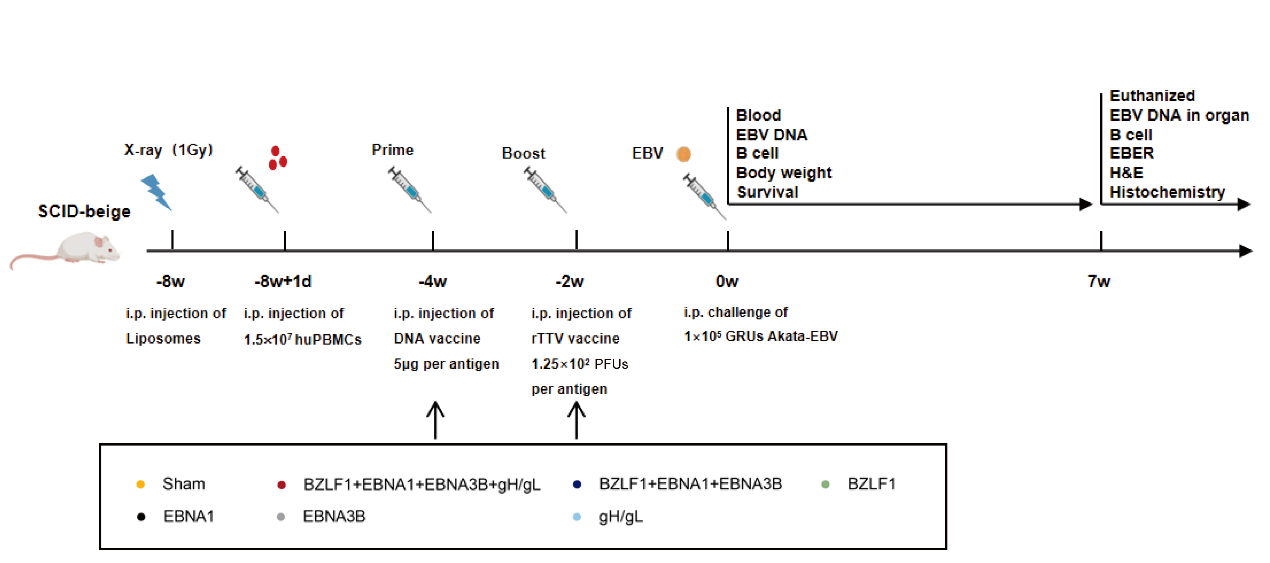


**Figure S14.** Experimental timeline

The figure was created from Biorender.com. Humanized mice were vaccinated with the multi-antigen vaccine expressing four antigens, the multi-antigen vaccine expressing BZLF1, EBNA1, and EBNA3B, and single-antigen DNA vaccines (5 μg per antigen) and rTTV vaccines (1.25×10^2^ PFUs per antigen) via intraperitoneal (i.p.) injection and heterologous prime-boost immunization schedules with a two-week interval. Humanized mice were bled and challenged with Akata EBV equivalent to 1×10^5^ Raji infectious units two weeks after the final immunization. Sham+EBV mice receive empty vector and virus, the multi-antigen vaccine expressing four antigens group n=6, the multi-antigen vaccine expressing BZLF1, EBNA1, and EBNA3B group n=5, all monovalent vaccine group n=5, and the sham group n=5.


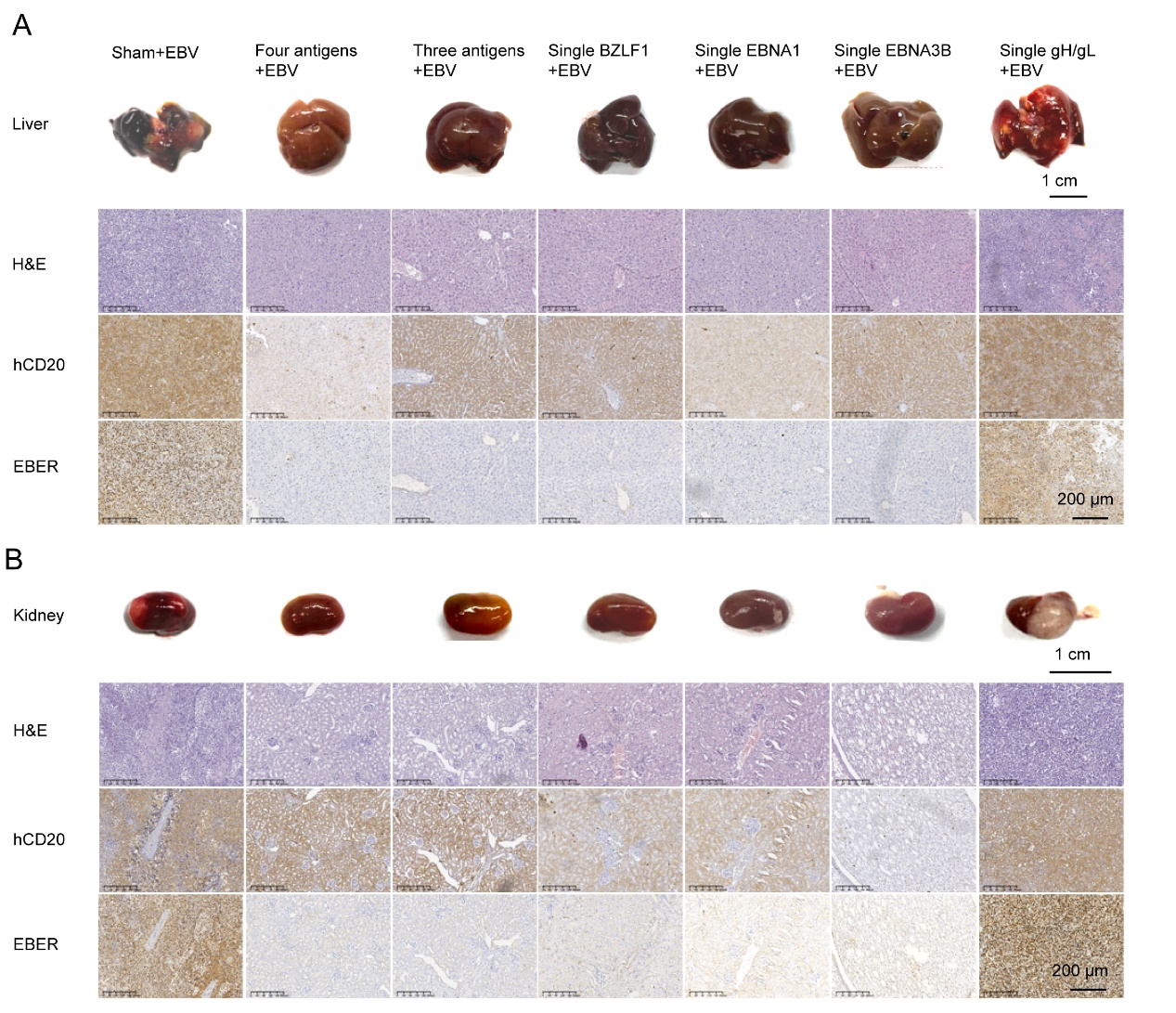


**Figure S15.** The multi-antigen vaccine expressing four antigens, the multi-antigen vaccine expressing BZLF1, EBNA1, and EBNA3B, the single-antigen BZLF1, the single-antigen EBNA1, and the single-antigen EBNA3B vaccine, except for the single-antigen gH/gL vaccine, reduce EBV replication in humanized mice

Representative of macroscopic liver (A) and kidney (B) sections stained for hematoxylin and eosin (H&E), human CD20 (hCD20), and EBV encoded RNA (EBER) at necropsy. Scale bar = 200 μm.

| Donor | A | B | C | DPB1 | DRB1 | DQB1 |
| --- | --- | --- | --- | --- | --- | --- |
| 1 | A*24:02:01;A*02:01:01 | B*55:02:01;B*15:12:01G | C*12:03:01;C*03:03:01 | DPB1*05:01:01;DPB1*02:01:02 | DRB1*14:05:01;DRB1*12:02:01 | DQB1*03:01:01;DQB1*05:03:01 |
| 2 | A*02:03:01G;A*02:03:01G | B*38:02:01G;B*15:02:01 | C*07:02:01;C*08:01:01 | DPB1*05:01:01;DPB1*21:01:01G | DRB1*16:02:01;DRB1*12:02:01 | DQB1*03:01:01;DQB1*05:02:01 |
| 3 | A*24:02:01;A*02:03:01G | B*52:01:01;B*40:01:02 | C*12:02:02;C*03:17:01 | DPB1*14:01:01;DPB1*04:01:01 | DRB1*16:02:01;DRB1*14:04:01G | DQB1*05:03:01;DQB1*05:02:01 |
| 4 | A*02:07:01;A*30:01:01 | B*40:01:02;B*37:01:01 | C*07:02:01;C*06:02:01 | DPB1*05:01:01;DPB1*05:01:01 | DRB1*10:01:01;DRB1*08:03:02 | DQB1*06:01:01;DQB1*05:01:01 |

**Table 1.** The Human Leukocyte Antigen (HLA) of human PBMCs donors.

Primer sequences of qRT-PCR (5′-3′).

| Gene name | Forward primer | Reverse primer |
| --- | --- | --- |
| BZLF1 | CTGGTGTCCGGGGGATAAT | TCCGCAGGTGGCTGCT |
| EBNA1 | GTTCCTCGCCTTAGGTTGTA | AGCTCTCCTGGCTAGGAGTC |
| EBNA3B | GGATCGTCACCACCATTGT | GGTGGGATCTGAGCCTATTT |
| BKRF2 (gL) | TCTCCATCCTGAAGCGAAGC | TGGCACCAAACAGGTCTTCC |
| BXLF2 (gH) | CCAGCACCACCTATCTCAGC | CAGGATTTCTGCGTCCTGGT |
| GAPDH | GCACCGTCAAGGCTGAGAAC | TGGTGAAGACGCCAGTGGA |

**Table 2** Primer sequences of qRT-PCR
